# Axial functionalisation of photoactive diazido platinum(iv) anticancer complexes

**DOI:** 10.1039/D0QI00685H

**Published:** 2020-08-26

**Authors:** Huayun Shi, Cinzia Imberti, Guy J. Clarkson, Peter J. Sadler

**Affiliations:** Department of Chemistry, University of Warwick, Coventry CV4 7AL, UK

## Abstract

Mono-axial functionalised octahedral diazido Pt(iv) complexes trans, trans, trans-[Pt(py)_2_(N_3_)_2_(OR_1_)(OR_2_)] (OR_1_ = OH and OR_2_ = anticancer agent coumarin-3 carboxylate (cou, **2a**), pyruvate dehydrogenase kinase (PDK) inhibitors 4-phenylbutyrate (PhB, **2b**) or dichloroacetate (DCA, **2c**)), and their di-axial functionalised analogues with OR_1_ = DCA and OR_2_ = cou (**3a**), PhB (**3b**), or DCA (**3c**) have been synthesised and characterised, including the X-ray crystal structures of complexes **2a, 3a, 3b** and **3c**. These complexes exhibit dark stability and have the potential to generate cytotoxic Pt(ii) species and free radicals selectively in cancer cells when irradiated. Mono-functionalised complexes **2a–2c** showed higher aqueous solubility and more negative reduction potentials. Mono- and di-functionalised complexes displayed higher photocytotoxicity with blue light (1 h, 465 nm, 4.8 mW cm^–2^) than the parent dihydroxido complex 1 (OR_1_ = OR_2_ = OH) in A2780 human ovarian (IC_50_ 0.9–2.9 μM for **2a–2c**; 0.11–0.39 μM for **3a–3c**) and A549 human lung cancer cells (5.4–7.8 μM for **2a–2c**; 1.2–2.6 μM for **3a–3c**) with satisfactory dark stability. Notably, no apparent dark cytotoxicity was observed in healthy lung MRC-5 fibroblasts for all complexes (IC_50_ > 20 μM). Significantly higher platinum cellular accumulation and photo-generated ROS levels were observed for the di-functionalised complexes compared with their mono-functionalised analogues when cancer cells were treated under the same concentrations.

## Introduction

Octahedral Pt(iv) complexes with low-spin 5d^6^ configurations are generally very stable, but can often be activated by bio-reductants *in vivo* (*e.g.* GSH, ascorbic acid, and cysteine residues) to give the corresponding more reactive Pt(ii) drugs.^1–3^ The Pt(iv) drugs iproplatin,^4,5^ ormaplatin,^6^ satraplatin^7^ and LA-12^8^ have entered clinical trials, but none has yet received clinical approval.^9^ A problem for Pt(iv) drugs, which are activated by chemical reduction in the body, is that the extent and cell selectivity of activation are difficult to control since they rely on natural processes.^2,3^ The use of localised light to control the activation of Pt(iv) prodrugs might overcome this problem.^10–12^

In contrast to Pt(iv) complexes which exert their cytotoxicity *via* chemical reduction, photoactive Pt(iv) complexes require higher dark stability and resistance to bio-reductants to guarantee that these prodrugs reach cancer cells and can be selectively photoactivated there.^2,10–14^ *Trans, trans, trans*-[Pt(py)_2_(N_3_)_2_(OH)_2_] (**1**) is a promising photoactive diazido Pt(iv) complexes that exhibits high dark stability and photoactivation upon visible light irradiation.^15^ The axial ligands of Pt(iv) complexes can be released during reduction, and greatly affect the reduction potential of Pt(iv).^16,17^ in general, Pt(iv) complexes with axial hydroxide ligands exhibit higher dark stability than those with other axial ligands.^18,19^ However, Pt(iv) prodrugs with bioactive axial ligands can selectively target cancer cells and attack several cellular components at the same time, providing a multi-targeted mechanism of action, which can result in enhanced anticancer potency and circumvention of cisplatin resistance.^3,20,21^ Here, conjugates of **1** with various anticancer drugs and cancer-targeting vectors as axial substituents have been investigated. Related reported complexes mostly have only one axial position derivatised and retain one hydroxide ligand to stabilise the Pt(iv) and increase aqueous solubility.^10^ Only one di-functionalised photoactive trans-diazido Pt(iv) complex with different functional ligands appears to have been reported so far.^22^

**Scheme 1:**
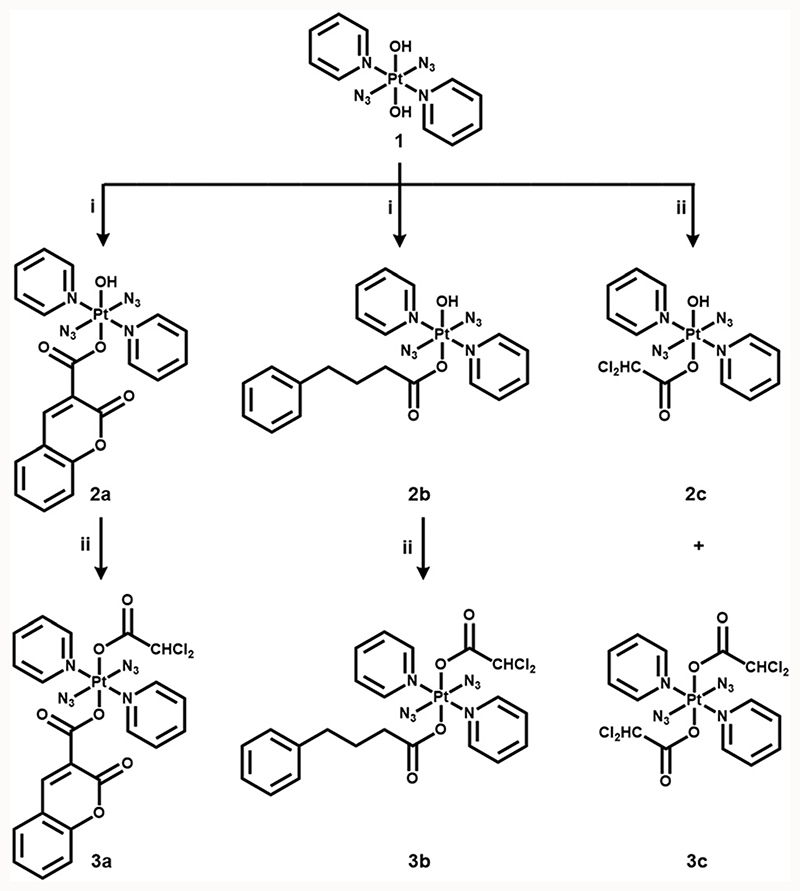
Synthetic routes for photoactive diazido Pt(iv) complexes **2a–2c** and **3a–3c**. (i) cou/PhB acid, TBTU, DIPEA, DMF, N_2_, RT, overnight; (ii) DCA anhydride, DMF, N_2_, RT, overnight.

The formation of esters *via* reactions of carboxylic acids and an axial hydroxide ligand in Pt(iv) complexes is a promising strategy for the development of multifunctional drugs.^3,10^ in this work, anticancer agent coumarin-3 carboxylate (cou), pyruvate dehydrogenase kinase (PDK) inhibitor 4-phenylbutyrate (PhB) and dichloroacetate (DCA) were conjugated to diazido Pt(iv) complex **1** to generate mono-functionalised complexes **2a–2c**, which were further modified with a DCA ligand to give di-functionalised complexes **3a–3c** (Scheme 1).

Coumarin and derivatives containing a benzopyrone target a number of pathways in cancer cells (*e.g*. kinase inhibition, cell cycle arrest, and angiogenesis inhibition) and have been widely used as anticancer agents.^23–27^ Coumarin derivatives can act as an antenna for light-harvesting when conjugated to metal complexes, and therefore enhance the fluorescence quantum yield and lengthen their emission lifetimes.^28^ PDK inhibitor PhB can suppress aerobic glycolysis, reverse the Warburg effect, and eventually kill cancer cells.^29^ In addition, PhB also acts as a weak extracellular histone deacetylase (HDAC) inhibitor.^29,30^ DCA is frequently tagged on to anticancer metal complexes for its mitochondrial targeting and PDK-inhibiting properties.^29,31^ The di-DCA Pt(iv) complex mitaplatin is activated by chemical reduction and then (i) liberates Pt(ii) species that can attack nuclear DNA and (ii) DCA that can attack mitochondria, giving rise to notable selectivity towards cancer cells.^31^ The combination of DCA and other functional substituents in a Pt(iv) complex can result in significantly improved _cytotoxicity._^3,22,29,31,32^

Photoactive complexes **2a–2c** and **3a–3c** with the general formula *trans, trans, trans*-[Pt(py)_2_(N_3_)_2_(OR_1_)(OR_2_)] have been synthesised and characterised, and the X-ray crystal structures of **2a, 3a, 3b** and **3c** were determined. The reduction potentials, dark stability and photodecomposition, photoreactions with 5′-GMP, photocytotoxicity and cellular accumulation are compared between mono-functionalised **2a–2c**, di-functionalised **3a–3c**, and unfunctionalised parent complex **1**.

## Results and discussion

### Synthesis and characterisation

The synthetic routes for photoactive Pt(iv) complexes **2a–2c** and **3a–3c** are summarised in Scheme 1. Mono-functionalised complexes **2a** and **2b** were obtained by combining parent complex **1** with the corresponding acid using TBTU as a coupling agent. The bi-functionalised complexes **3a** and **3b** were synthesised by stirring **2a** and **2b** with dichloroacetic anhydride, respectively, while **2c** and **3c** were made by reacting **1** directly with dichloroacetic anhydride. Complexes **2a–2c** and **3a–3c** are air-stable at 298 K in the solid state in the dark. Dark stability was observed for **2a–2c** in RPMI-1640 cell culture medium by UV-vis spectroscopy (Fig. S1, ESI†). Complexes **3a–3c** showed poor aqueous solubility, but were stable in DMSO (Fig. S1, ESI†). The dark stability of complexes 2a and 3a in the presence of 2 mM GSH was confirmed by UV-vis spectroscopy (Fig. S2, ESI†). All complexes were characterised by ESI-HR-MS, NMR and UV-vis spectroscopy and their purity was determined by HPLC as >95% (Fig. S3–S16, ESI†).

All observed *m/z* values and the isotopic mass distribution patterns for Pt and Cl match well with calculated HR-MS spectra (Fig. S4, ESI†). All ^1^H and ^13^C NMR spectra are in agreement with the proposed structures for the complexes (Fig. S5–16, ESI†). The Pt-coordinated pyridine can be identified by ^1^H NMR doublets with ^195^Pt satellites at ca. 9.0 ppm, and the triplets at *ca.* 8.1 and 7.7 ppm and by ^13^C NMR resonances at ca. 150, 142 and 126 ppm assignable to the α, γ and β C–H groups, respectively, of pyridine. The electronic absorption spectra of complexes **2a–2c** and **3a–3c** are similar to that of parent complex **1** with a maximum absorption band at ca. 300 nm (*ε ca.* 17 000 M^–1^ cm^–1^) assignable to LMCT (N_3_ → Pt) transitions. The extinction coefficients of 2a and 3a at ca. 300 nm (*ε ca*. 31 000 M^–1^ cm^–1^) are much higher due to the presence of the coumarin moiety.

### X-ray crystallography

**Fig. 1:**
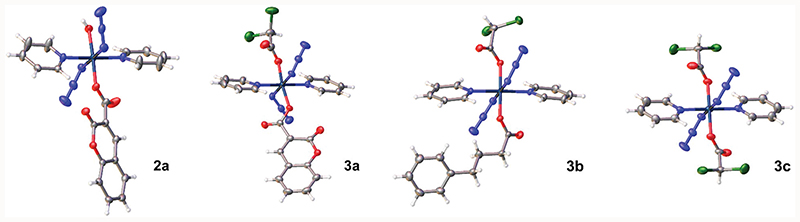
X-ray crystal structures of mono-functionalised **2a** and di-functionalised **3a, 3b**, and **3c**, with thermal ellipsoids drawn at 50% probability level.

Crystals of complexes **2a, 3a, 3b** and **3c** suitable for X-ray diffraction studies were obtained through evaporation, or diffusion of diethyl ether into DCM/MeOH solutions. The perspective drawings of complexes **2a** and **3a–3c** are shown in Fig. 1. The crystallographic data are summarised in Table S1 (ESI†) and selected bond distances and angles are listed in Tables 1 and S2 (ESI†). Complexes **2a, 3b** and **3c** crystallised in the triclinic $P\bar{1}$ space group, while complex **3a** crystallised in the trigonal $R\bar{3}$ space group. These complexes show similarities in the equatorial plane defined by four nitrogen atoms, two from the *trans* pyridine molecules and two from the *trans* azide anions, and resemble typical equatorial planes in their analogues.^15^ With azide-Pt bond angles of *ca.* 116°, the plane contains only the bound nitrogens of the azide ligands with the remainder of the azide ligands projected out on opposite faces of the plane. The difference lies in the axial ligands. Complex 2a is mono-functionalised with an O-bound coumarin-3 carboxylate and a *trans* hydroxide ligand (O–Pt–O bond angle of 175.13 (6)°), which forms a moderately strong hydrogen bond with OH in an adjacent molecule (O3–H3B⋯O3, Table S3, ESI†). For complex **3c** with two dichloroacetate ligands, the Pt sits on a crystallographic inversion centre, while complexes **3a** and **3b** exhibit distortions from ideal octahedral geometry, with *trans* O–Pt–O bond angles smaller than 180° (171.97(12)° for 3a, and 178.76(10)° for **3b**). The Pt–O bond distance is 2.005(3) Å for the coumarin-3 carboxylic acetate in complex **3a** and 1.992(3) Å for 4-phenylbutyrate in 3b. These distances are slightly shorter than the Pt–O bond lengths for their respective dichloroacetate ligands (2.020(3) and 2.016(3) Å).

### Cyclic voltammetry

**Fig. 2:**
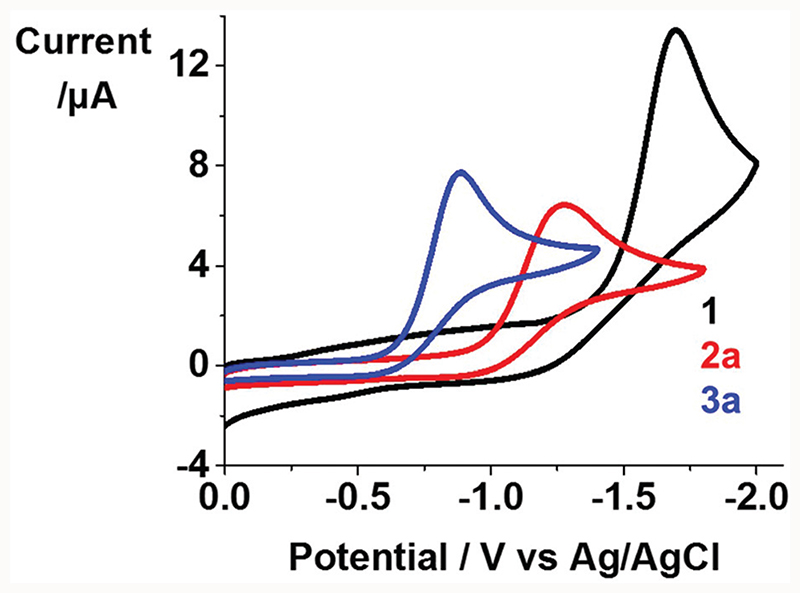
Cyclic voltammograms for complexes **1, 2a and 3a** (1 mM) in 0.1 M NBu_4_PF_6_-DMF (deaerated under N_2_).

Cyclic voltammograms for **1, 2a** and **3a** were acquired in the potential range –1.8–0.0 V in DMF at 298 K, using 0.1 M NBu_4_PF_6_ as supporting electrolyte (Fig. 2). An irreversible reduction wave assigned to Pt^IV^/Pt^II^ was observed with *E*_pc_ of –1.699, –1.285 and –0.886 V for **1, 2a** and **3a**, respectively. it is notable that Pt(iv) complexes with the more unmodified hydroxide axial ligands (OH) exhibit more negative reduction potentials, consistent with the ability of axial hydroxide to stabilise these Pt(iv) complexes.^18,19^ The irreversible reduction waves suggest that these complexes release ligands during reduction, as observed upon irradiation where cytotoxic species are released as a result of photoreduction.

### Photoactivation and radical formation

**Fig. 3:**
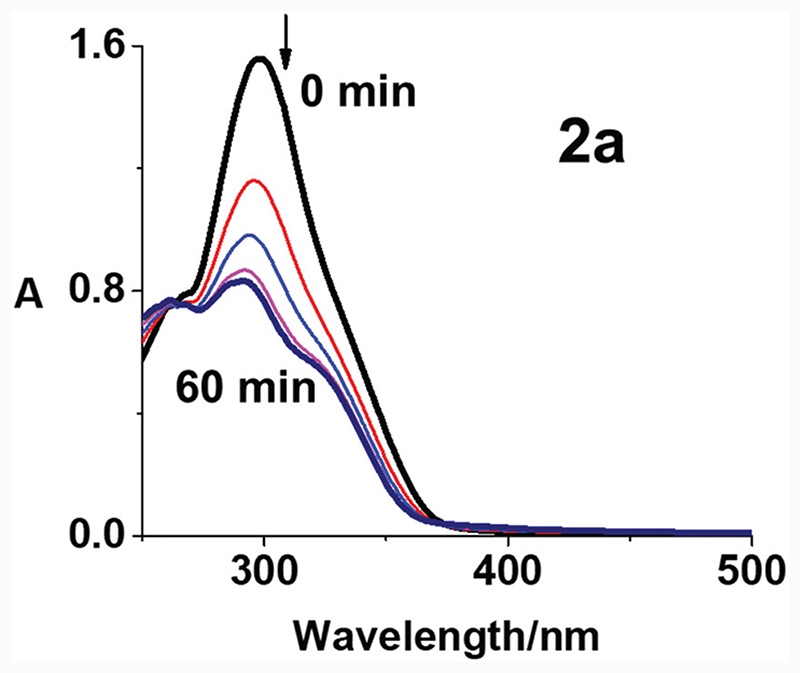
Photochemical decomposition of mono-functionalised Pt(iv) complex **2a** (50 μM) in phenol red-free RPMI-1640 cell culture medium with 5% DMSO (v/v) upon irradiation with blue light (420 nm, 1 h) at 298 K.

The photodecomposition of complexes **2a–2c** in RPMI-1640 ceII culture medium with 5% DMSO (used for solubilisation during cytotoxicity screening) and **3a–3c** in DMSO was monitored by UV-vis spectroscopy at different time intervals after irradiation with blue light (420 nm) at 298 K (Fig. 3, S17 and S18, ESI†). Fig. 3 shows the decrease in absorbance maximum of complex **2a** at 298 nm, assigned as a LMCT band (N_3_ → Pt). The decrease was rapid over the first 15 min and showed no significant further change after 30 min, suggesting release of the azide ligands. Similar results were obtained using mono-functionalised **2b** and **2c** (Fig. S17, ESI†), consistent with previously published data for complex **1**.^15^ Notably, the photodecomposition of di-functionalised **3a–3c** in DMSO was complete in 10 min with blue light irradiation (Fig. S18, ESI†). The incorporation of the coumarin-3 carboxylate provides the possibility of using complex 2a in fluorescence emission spectroscopic studies. Even though coumarin-3 carboxylic acid exhibited no apparent fluorescence in aqueous solution with excitation at 405 nm, it can exhibit switched-on blue fluorescence (ca. 450 nm) in the presence of OH^∂^, owing to the generation of fluorescent 7-hydroxycoumarin-3 carboxylic acid (p*K*_a_ = 1.98).^33^ While no apparent fluorescence was observed before irradiation, 2a exhibited emission centred at *ca*. 440 nm after irradiation with blue light (420 nm, 20 min) at 298 K (Fig. S19, ESI†). This emission can be attributed to the formation of 7-hydroxycoumarin-3 carboxylate from coumarin-3 carboxylate and OH^•^ radicals as photoproducts of **2a** which contains coumarin-3 carboxylate and hydroxide ligands. In contrast, no emission was detected for **3a** (which has no hydroxide ligands) under the same irradiation conditions. However, 7-hydroxycoumarin-3 carboxylate was not detected by LC-MS (Table S4, ESI†).

The photoproducts from complex **2a** irradiated in aqueous solution were investigated by LC-MS. Only a HPLC peak assigned to **2a** was observed before irradiation, indicating the high purity of the complex (>99%). This peak disappeared within 20 min of irradiation with blue light (420 nm, Fig. S20, ESI†). The peak assigned to {Pt^IV^(py)_2_(N_3_)(OH)(cou)}^+^ (601.18 *m*/*z*, peak **f** in Fig. S20 and Table S4, ESI†) increased in intensity up to 5 min, and thereafter decreased in 5–30 min with a concomitant increase in intensity of peaks for the photoproducts [2{Pt^II^(py)(OH)_2_(HCOO)} + 3Na]^+^ (774.99 *m/z*, **a**), {Pt^II^(py)(cou)(CH_3_CN)_2_}+ (545.10 *m/z*, **b**), {Pt^III^(py)_2_(HCOO) (N3)}^+^ (440.07 *m*/*z*, **c**), {Pt^II^(py)_2_(N_3_)(CH_3_CN)}^+^ (436.08 *m*/*z*, **d**), [{Pt^II^(py)_2_(N_3_)_2_} + Na]^+^ (460.08 *m/z*, **g**), [{Pt^II^(py)(cou)(HCOO) (N_3_)} + 2H]+ (552.05 *m/z*, **h**), and coumarin-3 carboxylic acid (191.29 *m*/*z*, **e** Fig. S20 and Table S4, ESI†). The formic acid and acetonitrile ligands arise from the mobile phase in LC-MS. The photodecomposition of **2a** is complicated. In general, one azide ligand appears to be released from Pt(iv) complexes first to form {Pt^IV^(py)_2_(N_3_)(OH)(cou)}^+^ (601.18 *m*/*z*, **f**), which can then liberate the other azide or the axial ligand (OH or cou) to form Pt(ii) species. When the irradiation time increased, some Pt(ii) species underwent further release of both axial ligands.

**Fig. 4:**
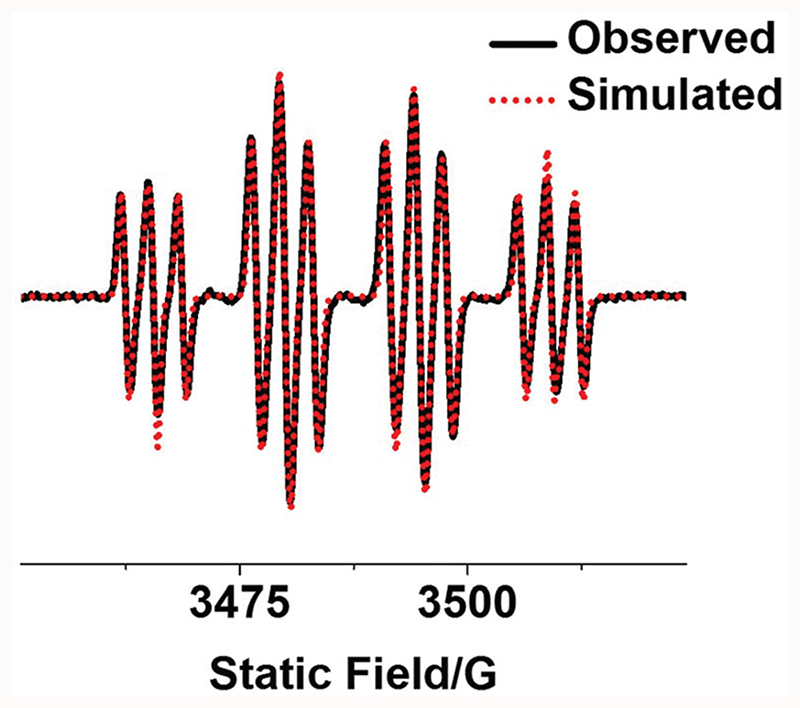
Observed (black) and simulated (red) EPR spectra of mono-functionalised complex **2a** (2.5 mM) with an axial coumarin in aqueous solution with 5% DMSO showing the formation of DMPO-N_3_^•^ and DMPO-OH^•^ adducts after irradiation (463 nm). The experimental trace is the accumulation of 300 scans with continuous irradiation (465 nm, 50 min). Parameters for simulation: DMPO-N_3_^•^ (*g* = 2.00595, $a_{\text{NO}}^{\text{N}}=1.43\;\text{mT}$, $a_{\beta }^{\text{H}}=1.45\;\text{mT}$ and $a_{\text{N}a}^{\text{N}}=0.29\;\text{mT}$); DMPO-OH^•^ (*g* = 2.00592, $a_{\text{NO}}^{\text{N}}=1.43\;\text{mT}$, and $a_{\beta }^{\text{H}}=1.45\;\text{mT}$.^34^

Reactive radicals released by photo-irradiated complex **2a** in aqueous solution were detected by EPR using the spin-trap 5,5-dimethyl-1-pyrroline-*N*-oxide (DMPO, Fig. 4). The EPR spectra for **2a** showed no signal in the absence of irradiation, while a 1: 2 : 2 : 1 quartet of triplet peaks assigned as DMPO-N_3_^•^ and a quartet signal as DMPO-OH^•^ were detected after irradiation (463 nm). Hence the EPR results suggest the formation of OH^•^ and N_3_^•^ radicals after irradiation (50 min).

### Photoreaction with 5′-GMP (guanosine 5′-monophosphate)

The photoreaction was carried out by irradiating an aqueous solution of complex **2a** (30 μM) in the presence of 2 mol equiv. of 5’-GMP with blue light (420 nm) at 310 K without prior incubation, and was monitored by LC-MS at different time intervals (Fig. S21, ESI†). The major photoproduct of the reaction was assigned as {Pt^II^(CH_3_CN)(py)_2_(GMP-H)}^+^ (756.16 *m/z*, G1), which increased in concentration with the irradiation time. Pt-GMP adducts {Pt^II^(HCOO)(py)_2_(GMP)}^+^ (762.10 *m/z*, G2) and {Pt^II^(N_3_)(py)_2_(GMP)}^+^ (758.16 *m/z*, G3) were also detected (formic acid and acetonitrile arise from the HPLC mobile phase). Notably, the major Pt-GMP adducts generated by **2a** are the same as those for **1** and its derivatives, indicating that the axial substituents do not affect the interaction between guanine and the platinum centre upon irradiation.^15,34^

### Photocytotoxicity and cellular accumulation studies

The photocytotoxicity of complexes **2a–2c** and **3a–3c** was determined in A2780 ovarian and A549 lung cancer cells, after 1 h drug incubation, 1 h irradiation (465 nm, 4.8 mW cm^–2^) and 24 h recovery (Table 2). Their dark cytotoxicity was also determined after 2 h incubation and 24 h recovery in A2780 and A549 cancer cells, as well as MRC-5 normal lung fibroblasts for comparison. Due to the poor aqueous solubility of these compounds, DMSO was used to facilitate their dissolution. Even though DMSO is a strong ligand for Pt(ii), no Pt-DMSO adduct was detected during irradiation (Table S4, ESI†). Also, due to the low DMSO concentration (<0.5%) and short incubation time (1 h in the dark and 1 h irradiation), DMSO did not exhibit significant effects on the cytotoxicity of these complexes.

The IC_50_ values determined by the sulforhodamine B (SRB) colorimetric assay are summarised in Table 2. Mono-functionalised Pt(iv) complexes **2a–2c** were non-toxic in the dark with IC_50_ values >100 μM, while di-functionalised Pt(iv) complexes **3a–3c** were cytotoxic to A2780 ovarian cancer in the dark (IC_50_ values of 1.3–1.9 μM), but relatively non-toxic to A549 lung cancer and normal MRC-5 lung cells (IC_50_ values >20 μM). The selective toxicity towards ovarian cancer cells might be an advantage for use of these complexes as phototherapeutic prodrugs for topical application, especially considering their low cytotoxicity towards normal MRC-5 cells.

The photocytotoxicity of mono-functionalised complexes **2a–2c** in A2780 ovarian cancer cells (IC_50_ values of 0.9–2.9 μM) was >2.4× higher than that of unfunctionalised **1** (7.1 μM), and in A549 lung cancer cells (IC_50_ values of 5.4–7.8 μM) was >6.6× higher than that of **1** (51.9 μM). Moreover, di-functionalised Pt(iv) complexes **3a–3c** (IC_50_ values of 0.11–0.39 μM for A2780; 1.2–2.6 μM for A549) showed >3× enhanced photocytotoxicity compared with their mono-functionalised analogues **2a–2c** in both of the cancer cell lines tested. Notably, similar photocytotoxicity indices (PI) were found for mono-functionalised **2a–2c** and di-functionalised **3a–3c** in lung A549 cancer cells (Table 2), while >2–17× higher PI values were determined for **2a–2c** compared to their corresponding di-functionalised analogues in ovarian A2780 cancer cells, although **3a–3c** showed significantly higher photocytotoxicity in A2780 cells.

The cellular accumulation of complexes **2a–2c** and **3a–3c** was determined in A2780 ovarian cancer cells, and that of **2a** and **3a** also for A549 lung cancer cells at the same concentration (2 μM, Table 3). Mono-functionalised complexes exhibited >19× higher accumulation than unfunctionalised **1**, and significantly higher cellular accumulation was observed for di-functionalised complexes compared to the corresponding mono-functionalised analogues (*ca*. 3–18×, Table 3). The lipophilicity (based on the reversed-phase HPLC retention time) has a major influence on cellular Pt accumulation, following the trend: di-functionalised **3a–3c** > mono-functionalised **2a–2c** > unfunctionalised **1** (Table 3). This trend matches that for photocytotoxicity well: di-functionalised **3a–3c** > mono-functionalised **2a–2c** > unfunctionalised **1**. These results suggest that the extent of cellular accumulation of these Pt(Iv) prodrugs plays an important role in their antiproliferative potency.

### Cellular ROS generation

**Fig. 5:**
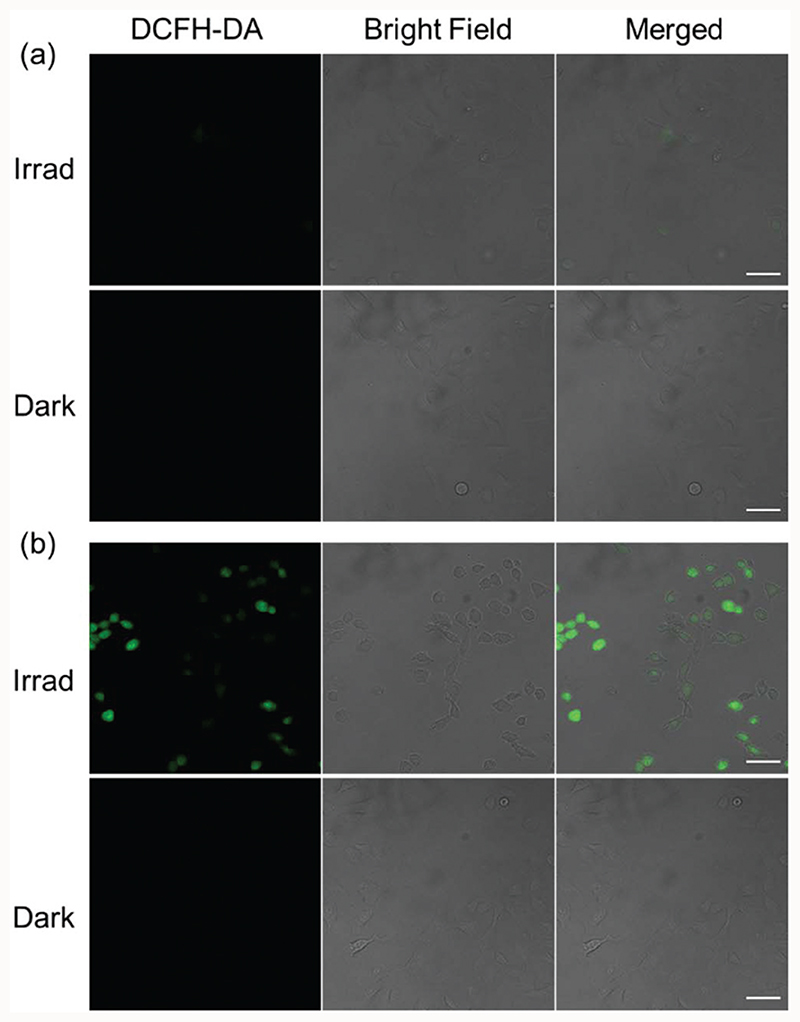
Confocal fluorescence microscopy images of ROS generation in A549 cells treated with (a) **2c** and (b) **3c** (2 μM, 1 h in dark and 1 h irradiation, 465 nm) then probed by DCFH-DA (20 μM, λ_ex_ = 488 nm). A549 cells treated in the dark were studied for comparison. Scale bar = 50 μm.

Since mitochondrial-targeting DCA can suppress aerobic glycolysis and increase mitochondrial oxidative phosphorylation and ROS production,^35^ the cellular ROS levels in A549 cells treated with mono-DCA complex **2c** or di-DCA complex **3c** were determined by the DCFH-DA assay (Fig. 5). DCFH-DA is a cell-permeable non-fluorescent probe that is converted to the highly fluorescent dye DCF upon oxidation by ROS in cells.^36^ As expected, A549 lung cancer cells treated with **3c** (2 μM) exhibited intense green fluorescence after 1 h irradiation (465 nm) indicative of ROS generation, while no fluorescence was observed in the dark. In contrast, cells treated with 2c (2 μM) displayed much less intense fluorescence after irradiation and again no fluorescence in the dark. These results indicate that a lower level of ROS was induced by mono-functionalised 2c compared to its di-functionalised analogue 3c, which correlates with its lower photocytotoxicity and cellular accumulation.

## Conclusions

Here we have synthesised and characterised six novel photoactive trans-diazido Pt(iv) dipyridine complexes, which differ in the presence of either one axial hydroxide ligand (mono-functionalised complexes **2a–2c**, with cou, PhB or DCA as an axial ligand, respectively), or no axial hydroxide ligands (di-functionalised complexes **3a–3c** with DCA as the second axial ligand). The X-ray crystal structures of complexes **2a** and **3a–3c** show a typical octahedral structure slightly distorted by the asymmetric axial ligands. These complexes are photoactive, generate Pt(ii) species which bind to 5′-GMP as monitored by LC-MS, and release azidyl and hydroxyl radicals as detected by EPR. Both axial ligands can be released from these complexes upon irradiation. However, mono-functionalised and di-functionalised complexes displayed significant differences in their photochemical and photobiological properties as summarised in Table 4.

Notably, mono-functionalised complexes **2a–2c** with one hydroxide ligand show higher aqueous solubility, and more negative reduction potentials. In contrast, due to the replacement of hydroxide with a lipophilic dichloroacetate ligand, di-functionalised complexes **3a–3c** exhibited much reduced aqueous solubility. Significantly higher cellular Pt accumulation (*ca*. 3–18×) and photo-induced cellular ROS levels were observed for di-functionalised complexes compared with their mono-functionalised analogues when cells were treated with complexes at the same concentration. As a result, higher photocytotoxicity (>3×) of di-functionalised complexes **3a–3c** compared with **2a–2c** and **1** in cancer cells was found. Mono-functionalised complexes **2a–2c** also showed improved photocytotoxicity (>2.4× in A2780; >6.6× in A549) compared to unfunctionalised **1** and were non-toxic in the dark (IC_50_ values >100 μM). Notably, all of these complexes showed a higher photocytotoxicity and cellular accumulation in A2780 ovarian cancer cells compared to A549 lung cancer cells, and low cytotoxicity towards normal MRC-5 cells. Mono-functionalised complexes exhibited higher photoselectivity towards A2780 cells due to their low dark cytotoxicity, but di-functionalised complexes exhibited a selectivity towards A2780 cells both in the dark and upon irradiation with extremely high cellular accumulation and high potency (Iow-dose required). Thus, these di-functionalised complexes are promising candidates as clinical prodrugs to treat ovarian cancer locally by phototherapy.

## Supplementary Material

ESI

## Figures and Tables

**Table 1:** Selected bond lengths (Å) and bond angles (°) for **2a** and **3a**

Complex 2a		Complex 3a	
Pt1–O3	1.9753(17)	Pt1–O19	2.020(3)
Pt1–N25	2.049(2)	Pt1–N13	2.045(4)
Pt1–N28	2.044(2)	Pt1–N16	2.058(4)
Pt1–O1	2.0289(16)	Pt1–O24	2.005(3)
Pt1–N13	2.033(2)	Pt1–N1	2.040(3)
Pt1–N19	2.025(2)	Pt1–N7	2.028(3)
N25–N26	1.212(3)	N13–N14	1.211(5)
N26–N27	1.139(3)	N14–N15	1.147(5)
N28–N29	1.209(3)	N16–N17	1.222(5)
N29–N30	1.147(3)	N17–N18	1.148(6)
O1–Pt1–O3	175.13(6)	O19–Pt1–O24	171.97(12)
N13–Pt1–N19	178.33(8)	N1–Pt1–N7	179.26(15)
N25–Pt1–N28	179.07(8)	N13–Pt1–N16	178.49(14)
N26–N25–Pt1	116.89(17)	N14–N13–Pt1	116.0(3)
N25–N26–N27	174.3(3)	N13–N14–N15	175.2(5)
N29–N28–Pt1	117.37(18)	N17–N16–Pt1	114.9(3)
N28–N29–N30	174.6(3)	N16–N17–N18	175.2(4)
		CI23–C21–CI22	112.1(3)

**Table 2:** IC_50_ values and photocytotoxicity indices (PI) for mono-functionalised complexes **2a–2c** and di-functionalised **3a–3c** for A2780 ovarian and A549 lung cancer cells, and MRC-5 normal lung fibroblasts (1 h incubation, 1 h irradiation (465 nm), followed by 24 h recovery). Data for unfunctionalised complex **1** are listed for comparison^34^

Complex		IC_50_^*a*^ (μM)		
		A2780	A549	MRC5
2a	Dark^*b*^	>100	>100	>100
	Irrad	2.9 ± 0.2	7.8 ± 0.1	
	PI	>34	>12	
2b	Dark	>100	>100	>100
	Irrad	0.92 ± 0.07	5.44 ± 0.05	
	PI	>108	>18	
2c	Dark	>100	>100	>100
	Irrad	1.2 ± 0.2	6.6 ± 1.1	
	PI	>83	>15	
3a	Dark	1.9 ± 0.1	>50	>50
	Irrad	0.11 ± 0.02	2.6 ± 0.3	
	PI	17.3	>19	
3b	Dark	1.3 ± 0.2	>20	>20
	Irrad	0.15 ± 0.01	1.2 ± 0.1	
	PI	8.7	>16	
3c	Dark	1.9 ± 0.3	>20	>20
	Irrad	0.39 ± 0.01	1.9 ± 0.1	
	PI	4.9	>10	
1	Dark	>100	>100	>100
	Irrad	7.1 ± 0.4	51.9 ± 2.5	
	PI	>14	>1.9	

a Each value is the mean of two independent experiments ± standard deviation.

b Dark cytotoxicity was determined after 2 h incubation and 24 h recovery.

**Table 3:** Cellular accumulation of Pt in A2780 cells and A549 cells after exposure to unfunctionalised 1, mono- and di-functionalised complexes **2a–2c** and **3a–3c**, respectively (2 μM, 1 h, in the dark)

Cell	Complex	Pt^*a*^ (ng per 10^6^ cells)
A549	**1**	0.51 ± 0.03***
	**2a**	9.8 ± 2.1*
	**3a**	36.1 ± 1.5***
A2780	**1**	0.16 ± 0.02*
	**2a**	6.2 ± 0.5**
	**2b**	3.5 ± 0.4***
	**2c**	10.8 ± 0.3***
	**3a**	90.9 ± 12.0***
	**3b**	61.4 ± 5.0***
	**3c**	53.8 ± 1.9***

a All data were determined from triplicate samples and their statistical significance compared to untreated cells evaluated by a two-tail *t*-test with unequal variances. *p < 0.05, **p < 0.01, ***p < 0.005.

**Table 4:** Comparison between mono- and di-functionalised complexes studied in this work, with dihydroxido complex **1** as a reference

Property	Mono-functionalised **2a–2c**	Di-functionalised **3a–3c**
Aqueous solubility	Good	Poor
Reduction potential	More negative	Less negative
Dark cytotoxicity	Similar to **1** (low)	Higher
Photo-cytotoxicity	Higher than **1**	Higher than **2a–2c**
Cellular accumulation	Higher than **1**	Much higher than **2a–2c**
ROS generation	Low	High
