# Supplementary material for: Axial functionalisation of photoactive diazido platinum(iv) anticancer complexes
Source: Inorg Chem Front. Author manuscript; Available in PMC 2021 Mar 29. (PMC7610473; doi:10.1039/D0QI00685H)
Supplement: ESI [file EMS119414-supplement-ESI.pdf]

Electronic Supplementary Material (ESI) for Inorganic Chemistry Frontiers.
This journal is © the Partner Organisations 2020

Electronic Supporting Information

Axial functionalisation of photoactive diazido platinum(IV) anticancer complexes

Huayun Shi, Cinzia Imberti, Guy Clarkson, Peter J Sadler*

Department of Chemistry, University of Warwick, Coventry CV4 7AL, UK

Experimental section 2
Table S1. Crystal data and structure refinement for **2a**, **3a**, **3b** and **3c**. 9
Table S2. Selected bond lengths (Å) and bond angles (°) for **3b** and **3c**. 10
Table S3. Selected hydrogen bonds parameters for **2a**. 10
Table S4. Photochemical decomposition products of **2a** detected by LC-MS. 10
Figure S1. Dark stability of **2a**–**2c** in phenol red-free RPMI-1640 and **3a**–**3c** in DMSO. 11
Figure S2. Dark stability in the presence of GSH of **2a** in H_2O and **3a** in DMSO. 12
Figure S3. HPLC purity of **2a**–**2c** and **3a**–**3c** with detection wavelength 254 nm. 12
Figure S4. HR-MS of complexes **2a**–**2c** and **3a**–**3c**. 13
Figure S5. 400 Hz ^{1}H NMR spectrum of complex **2a**. 14
Figure S6. 125 Hz DEPT-135 ^{13}C NMR spectrum of complex **2a**. 14
Figure S7. 500 Hz ^{1}H NMR spectrum of complex **3a**. 15
Figure S8. 125 Hz DEPT-135 ^{13}C NMR spectrum of complex **3a**. 15
Figure S9. 400 Hz ^{1}H NMR spectrum of complex **2b**. 16
Figure S10. 100 Hz DEPT-135 ^{13}C NMR spectrum of complex **2b**. 16
Figure S11. 400 Hz ^{1}H NMR spectrum of complex **3b**. 17
Figure S12. 100 Hz DEPT-135 ^{13}C NMR spectrum of complex **3b**. 17
Figure S13. 400 Hz ^{1}H NMR spectrum of complex **2c**. 18
Figure S14. 100 Hz DEPT-135 ^{13}C NMR spectrum of complex **2c**. 18
Figure S15. 400 Hz ^{1}H NMR spectrum of complex **3c**. 19
Figure S16. 125 Hz DEPT-135 ^{13}C NMR spectrum of complex **3c**. 19
Figure S17. Photochemical decomposition of **2b** and **2c** in RPMI-1640. 20
Figure S18. Photochemical decomposition of **3a**–**3c** in DMSO. 20
Figure S19. Fluorescence change during photochemical decomposition of complex **2a** in H_2O. 21
Figure S20. Photochemical decomposition of **2a** in aqueous solution monitored by HPLC. 21
Figure S21. Photoreaction between of **2a** and 5'-GMP in aqueous solution monitored by HPLC. 22

Experimental section

1. Materials and instruments

O-(Benzotriazol-1-yl)-*N*, *N*, *N*', *N*'-tetramethyluronium tetrafluoroborate (TBTU) was purchased from Merck; pyridine was from Fischer Scientific UK; 4-phenylbutyric acid was from Acros Organics; and other chemicals from Sigma Aldrich and used without further purification. Complex *trans*, *trans*, *trans*-[Pt(py)$_2$(N$_3$)$_2$(OH)$_2$] (**1**) was synthesised and characterised according to published procedures.[S1]

NMR spectra were recorded on Bruker Avance III 400 MHz, Bruker Avance III HD 400 MHz or Bruker Avance III HD 500 MHz spectrometers and referenced to the residual signal of the solvent. ESI-HR-MS data were collected on a Bruker microTOF instrument at 298 K with a scan range of m/z 50-2000 in positive mode. Samples were prepared in methanol solution. Electronic absorption spectra were recorded on a Varian Cary 300 UV-vis spectrophotometer in a 1-cm pathlength quartz cuvette with neat solvent as a reference. Fluorescence spectra were recorded in Jasco FP-6500 Spectrofluorometer. Analytical reversed-phase HPLC analyses were carried out on an Agilent ZORBAX Eclipse XDB-C18 column (250×4.6 mm, 5 µm, flow rate: 1 mL/min), by using linear gradients of 0.1% trifluoroacetic acid (TFA) in H_2O (solvent A) and 0.1% TFA in CH_3CN (solvent B, 10–80% in 30 min). LC-MS were carried out on Bruker Amazon X connected online with an Agilent 1260 HPLC, using 0.1% formic acid (FA) in H_2O (solvent A) and 0.1% FA in CH_3CN (solvent B, 10–80% in 30 min).

An LZC-ICH2 photoreactor (Luzchem Research Inc.) equipped with a temperature controller and 8 Luzchem LZC-420 lamps without light filtration, and LED light sources (BASETech model no. SP-GU10 230 V~50 Hz 1.3-2.1 W) with λ_{max} = 463 nm were used for photoactivation. An LED light source with λ_{max} = 465 nm (4.8 mW/cm^2) was used in *in vitro* growth inhibition assay.

The platinum content was analysed on an ICP-OES 5300DV (Perkin Elmer, λ_{em} = 265.945 nm) or ICP-MS 7500cx (Agilent, ^{195}Pt). Samples were calibrated with standards prepared from a stock 1000 ppm Pt solution obtained from Sigma Aldrich in 3.6% HNO_3 and Milli-Q water.

2. Synthesis and characterisation

Caution! Even though we encountered no accidents, heavy metal azides can be shock-sensitive and should be handled with due care and attention in the dark.

Trans, trans, trans-[Pt(py)$_2$(N$_3$)$_2$(cou)(OH)] (**2a**). To the solution of complex **1** (40.0 mg, 85 μmolol), coumarin-3 carboxylic acid (16.2 mg, 85 μmol), and TBTU (27.3 mg, 85 μmol) in DMF solution (2 mL), N, N-diisopropylethylamine (DIPEA) (100 μL) was added. The reaction mixture was stirred overnight at 298 K under a nitrogen atmosphere. After evaporation to dryness, the oily residue was collected and purified by column chromatography on silica gel (3% methanol + 97% DCM). Yield: 41 mg, 76%. ^{1}H NMR (CDCl$_3$, 400 MHz): 9.09 (d, J = 5.8 Hz, J^{195} Pt–^{1} H = 27.5 Hz, 4H, *H$_α$ py*), 8.33 (s, 1H, *CH$_{cou}$*), 8.12 (t, J = 7.7 Hz, 2H, *H$_γ$ py*), 7.61 (t, J = 6.9 Hz, 4H, *H$_β$ py*), 7.49 (t, J = 7.9 Hz, 1H, *H$_{cou}$*), 7.42 (d, J = 7.4 Hz, 1H, *H$_{cou}$*), 7.24 (d, J = 8.4 Hz, 1H, *H$_{cou}$*), 7.18 (t, J = 7.6 Hz, 1H, *H$_{cou}$*), 3.38 (s, 1H, *OH*). ^{13}C NMR (CDCl$_3$, 125 MHz): 167.10 (*COO*), 158.10 (*COO*), 154.90 (=*C*–), 149.87 (*C$_α$ py*), 146.97 (*CH*), 141.27 (*C$_γ$ py*), 133.17 (*CH*), 129.11 (*CH*), 125.95 (*C$_β$ py*), 124.43 (*CH*), 121.51 (=*C*–), 118.45 (=*C*–), 116.47 (*CH*). ESI-HR-MS: [M + Na]$^+$ (*m/z*) Calc., 666.0785; Found, 666.0777. $ε_{298\ nm}$ = 31660 M^{-1} cm^{-1}, $ε_{266\ nm}$ = 15847 M^{-1} cm^{-1} (5% DMSO + 95% RPMI-1640).

Trans, trans, trans-[Pt(py)$_2$(N$_3$)$_2$(cou)(DCA)] (**3a**). Three mol. equiv. of dichloroacetic anhydride were stirred with complex **2a** in DMF solution overnight at 298 K under a nitrogen atmosphere. After evaporation to dryness, the oily residue was collected and purified by column chromatography on silica gel (1% methanol + 99% DCM). Yield: 31 mg, 67%. ^{1}H NMR (CDCl$_3$, 500 MHz): 9.20 (d, J = 5.6 Hz, J^{195} Pt–^{1} H = 26.5 Hz, 4H, *H$_α$ py*), 8.32 (s, 1H, *CH$_{cou}$*), 8.14 (t, J = 7.6 Hz, 2H, *H$_γ$ py*), 7.72 (t, J = 7.1 Hz, 4H, *H$_β$ py*), 7.60 (t, J = 7.8 Hz, 1H, *H$_{cou}$*), 7.49 (d, J = 7.6 Hz, 1H, *H$_{cou}$*), 7.33 (d, J = 8.4 Hz, 1H, *H$_{cou}$*), 7.28 (t, J = 7.8 Hz, 1H, *H$_{cou}$*), 5.87 (s, 1H, *CHCl$_2$*). ^{13}C NMR (CDCl$_3$, 125 MHz): 167.46 (*COO*), 166.72 (*COO*), 157.68 (*COO*), 155.03 (=*C*–), 150.06 (*C$_α$ py*), 147.72 (*CH*), 141.76 (*C$_γ$ py*), 133.66 (*CH*), 129.28 (*CH*), 126.08 (*C$_β$ py*), 124.60 (*CH*), 119.63 (=*C*–), 118.14 (=*C*–), 116.55 (*CH*), 65.42 (*CHCl$_2$*). ESI-HR-MS: [M + Na]$^+$ (*m/z*) Calc., 776.0111; Found, 776.0115. $ε_{306\ nm}$ = 30650 M^{-1} cm^{-1} (DMSO).

Trans, trans, trans-[Pt(py)$_2$(N$_3$)$_2$(pb)(OH)] (**2b**). To the solution of complex **1** (40.0 mg, 85 μmol), 4-phenylbutyric acid (14.0 mg, 85 μmol), and TBTU (27.3 mg, 85 μmol) in DMF solution (2 mL), DIPEA (100 μL) was added. The reaction mixture was stirred overnight at 298 K under a nitrogen atmosphere. After evaporation to dryness, the oily residue was collected and purified by column chromatography on silica gel (2% methanol + 98% DCM). Yield: 47

mg, 89%. ^{1}H NMR ($CDCl_3$, 400 MHz): 8.95 (d, J = 5.3 Hz, J^{195} Pt–^{1} H = 27.3 Hz, 4H, H_α *py*), 8.06 (t, J = 7.6 Hz, 2H, H_γ *py*), 7.62 (t, J = 7.1 Hz, 4H, H_β *py*), 7.23 (d, J = 7.6 Hz, 2H, H_{phenyl}), 7.18-7.13 (m, 3H, H_{phenyl}), 3.46 (s, 1H, *OH*), 2.60 (t, J = 7.8 Hz, 2H, CH_2), 2.43 (t, J = 7.5 Hz, 2H, CH_2), 1.89 (m, 2H, CH_2). ^{13}C NMR ($CDCl_3$, 100 MHz): 177.13 (*COO*), 149.65 (C_α *py*), 142.08 (C_{phenyl}), 141.26 (C_γ *py*), 128.51 (C_{phenyl}), 128.24 (C_{phenyl}), 125.92 (C_β *py*), 125.71 (C_{phenyl}), 36.37 (CH_2), 35.24 (CH_2), 27.79 (CH_2). ESI-HR-MS: $[M + H]^+$ (*m/z*) Calc., 618.1537; Found, 618.1537. $\varepsilon_{297\ nm}$ = 19187 M^{-1} cm^{-1}, $\varepsilon_{259\ nm}$ = 13232 M^{-1} cm^{-1} (5% DMSO + 95% RPMI-1640).

Trans, trans, trans-[Pt(py)$_2$(N_3)$_2$(pb)(DCA)] (**3b**). Three mol. equiv. of dichloroacetic anhydride were stirred with complex **2b** in DMF solution overnight at 298 K under a nitrogen atmosphere. After evaporation to dryness, the oily residue was collected and purified by column chromatography on silica gel (1% methanol + 99% DCM). Yield: 30 mg, 63%. ^{1}H NMR ($CDCl_3$, 400 MHz): 8.92 (d, J = 6.0 Hz, J^{195} Pt–^{1} H = 24.8 Hz, 4H, H_α *py*), 8.10 (t, J = 7.5 Hz, 2H, H_γ *py*), 7.65 (t, J = 6.8 Hz, 4H, H_β *py*), 7.24 (d, J = 7.5 Hz, 2H, H_{phenyl}), 7.19-7.09 (m, 3H, H_{phenyl}), 5.83 (s, 2H, $CHCl_2$) 2.56 (t, J = 7.6 Hz, 2H, CH_2), 2.42 (t, J = 7.6 Hz, 2H, CH_2), 1.83 (m, 2H, CH_2). ^{13}C NMR ($CDCl_3$, 100 MHz): 178.26 (*COO*), 166.89 (*COO*), 149.59 (C_α *py*), 141.87 (C_γ *py*), 141.50 (C_{phenyl}), 128.44 (C_{phenyl}), 128.34 (C_{phenyl}), 126.23 (C_β *py*), 125.91 (C_{phenyl}), 65.50 ($CHCl_2$), 35.64 (CH_2), 35.00 (CH_2), 27.46 (CH_2). ESI-HR-MS: $[M + Na]^+$ (*m/z*) Calc., 750.0682; Found, 750.0673. $\varepsilon_{313\ nm}$ = 17654 M^{-1} cm^{-1} (DMSO).

Trans, trans, trans-[Pt(py)$_2$(N_3)$_2$(DCA)(OH)] (**2c**) and *trans, trans, trans*-[Pt(py)$_2$(N_3)$_2$(DCA)$_2$] (**3c**). Three mol. equiv. of dichloroacetic anhydride were stirred with complex **1** in DMF solution overnight at 298 K under a nitrogen atmosphere. After evaporation to dryness, the oily residue was collected and purified by column chromatography on silica gel (1% methanol + 99% DCM).

2c. Yield: 29 mg, 46%. ^{1}H NMR ($CDCl_3$, 400 MHz): 8.96 (d, J = 6.1 Hz, J^{195} Pt–^{1} H = 27.0 Hz, 4H, H_α *py*), 8.16 (t, J = 7.6 Hz, 2H, H_γ *py*), 7.72 (t, J = 7.0 Hz, 4H, H_β *py*), 5.96 (s, 1H, $CHCl_2$). ^{13}C NMR ($CDCl_3$, 100 MHz): 167.76 (*COO*), 149.30 (C_α *py*), 141.63 (C_γ *py*), 126.23 (C_β *py*), 66.80 ($CHCl_2$). ESI-HR-MS: $[M + Na]^+$ (*m/z*) Calc., 603.9950; Found, 603.9949. $\varepsilon_{299\ nm}$ = 16575 M^{-1} cm^{-1}, $\varepsilon_{259\ nm}$ = 11697 M^{-1} cm^{-1} (5% DMSO + 95% RPMI-1640).

3c. Yield: 21 mg, 28%. ^{1}H NMR ($CDCl_3$, 400 MHz): 8.96 (d, J = 5.3 Hz, J^{195} Pt–^{1} H = 25.6 Hz, 4H, H_α *py*), 8.14 (t, J = 7.6 Hz, 2H, H_γ *py*), 7.70 (t, J = 7.2 Hz, 4H, H_β *py*), 5.84 (s, 2H, *$CHCl_2$*). ^{13}C NMR ($CDCl_3$, 125 MHz): 167.46 (*COO*), 149.52 (C_α *py*), 142.10 (C_γ *py*), 126.37 (C_β *py*), 65.05 (*$CHCl_2$*). ESI-HR-MS: $[M + Na]^+$ (*m/z*) Calc., 713.9276; Found, 713.9273. $\varepsilon_{319\ nm}$ = 16257 M^{-1} cm^{-1} (DMSO).

3. X-Ray crystallography

Single crystals of **2a**, **3a**, **3b** and **3c** were grown by evaporation or diffusion of diethyl ether into corresponding DCM/MeOH solution at 298 K. A suitable crystal was selected and mounted on a glass fibre with Fromblin oil and placed on a Rigaku Oxford Diffraction SuperNova diffractometer with a duel source (Cu at zero) equipped with an AtlasS2 CCD area detector. The crystal was kept at 150(2) K during data collection. Using Olex2,[S2] the structure was solved with the ShelXT[S3] structure solution program using Direct Methods and refined with the ShelXL[S4] refinement package using Least Squares minimisation. CCDC 2003123−2003126 contain the supplementary crystallographic data for this paper.

4. Cyclic voltammetry

All cyclic voltammogram (CV) experiments were carried out using a CH Instrument model 600D Electrochemical Analyser/Workstation (Austin, TX). The solution was prepared in DMF containing tetrabutylammonium hexafluorophosphate (0.1 M) as supporting electrolyte and degassed under nitrogen. A typical three-electrode system was used to scan the cyclic voltammograms: a glassy carbon electrode as the working electrode, Ag/AgCl in 3.0 M KCl as the reference electrode (0.21 V versus NHE), and platinum wire as the counter electrode. The scan rate was set to be 100 mV/s.

5. Dark stability and photodecomposition

Complexes **2a**–**2c** were dissolved in 95% phenol red-free RPMI-1640 + 5% DMSO to test the dark stability monitored by UV-vis spectroscopy. The dark stability of complexes **3a**–**3c** was investigated in DMSO. The dark stability in the presence of 2 mM GSH of **2a** in 95% H_2O + 5% DMSO and **3a** in 95% DMSO + 5% H_2O (added to improve the solubility of GSH) was

monitored by UV-vis spectroscopy. The photodecomposition of complexes **2a**–**2c** in 95% phenol red-free RPMI-1640 + 5% DMSO and **3a**–**3c** in DMSO was monitored by UV-vis spectroscopy at different time intervals after irradiation with blue light (420 nm) at 298 K. The photodecomposition of complex **2a** in aqueous solution was also monitored by fluorescence spectroscopy and LC-MS (420 nm).

6. Electron paramagnetic resonance (EPR) spectroscopy

The EPR spectra were recorded on a Bruker EMX (X-band) spectrometer at 298 K. Samples (*ca*. 100 μL) in aqueous solution were prepared and transferred using a plastic syringe with metal needle to a standard quality quartz tube with inner diameter of 1.0 mm and outer diameter of 2.0 mm (Wilmad LabGlass) and sealed with parafilm. Typical key EPR spectrometer settings were modulation amplitude 1.0 G, microwave power 6.32 mW, 1.0×10^5 receiver gain, conversion time 5.12 ms, time constant 5.12 ms, sweep time 10.49 s for each scan, sweep width 200 G. The LED light (463 nm) source was mounted between the EPR magnets, supported by a foam sponge, to maintain its position throughout the EPR sample measurements. The distance from the tip of the irradiation light bulb to the EPR cavity was *ca*. 3 cm. Data were processed by Matlab R2016b with easyspin 5.1.12.

7. Photoreaction with 5'-GMP

The mixture of 2 mol. equiv. of guanosine 5′-monophosphate disodium salt hydrate (5′-GMP-Na_2) and 30 μM of each complex in aqueous solution was irradiated by blue light (420 nm) at different time intervals and analysed immediately by LC-MS.

8. Cell culture

Human cell lines, A2780 ovarian carcinoma, A549 lung adenocarcinoma cells and healthy lung MRC-5 fibroblasts were obtained from the European Collection of Animal Cell Culture (ECACC), Salisbury, UK. All cell lines used in this work were grown in Roswell Park Memorial Institute media (RPMI-1640), which was supplemented with 10% v/v of foetal calf serum (FCS) and 1% v/v penicillin/streptomycin. The adherent monolayers of cells were grown

at 310 K in a humidified atmosphere containing 5% CO_2 and passaged regularly at *ca*. 80% confluence.

9. Photo-dark cytotoxicity

Approximately 1.5×10^4 cells were seeded per well in 96-well plates. Independent duplicate plates were used, one for dark while the other for irradiation experiment. The cells were pre-incubated in drug-free medium with phenol red at 310 K for 24 h. Complexes were dissolved first in DMSO and then diluted in phenol red-free RPMI-1640 to make the stock solution of the drug. These stock solutions were further diluted using phenol-red free cell culture medium until working concentrations were achieved, the maximum DMSO concentration was < 0.5% v/v in these solutions. Cells were exposed to the complexes at various concentrations for 1 h. Then one plate was irradiated for 1 h using blue light (4.8 mW cm^{-2} per LED at 465 nm) while the dark plate was kept in the incubator. After irradiation, supernatants of both plates were removed by suction and the cells were washed with phosphate-buffered saline (PBS). Thus, cells were exposed to drugs and DMSO for only 2 h (1 h in the dark and 1 h upon irradiation) in total. Photocytotoxicity was determined after another 24 h recovery at 310 K in complex-free phenol red-containing medium by comparison to untreated controls which were exposed only to the vehicle. Untreated controls were also compared between the irradiated and the non-irradiated plates to ensure that the differences in cell survival were not statistically relevant, hence guaranteeing that the differences in cell viability observed were not caused by the light source. The cell viability was determined by the SRB assay.[S5] Absorbance measurements of the solubilised dye (on a Promega microplate reader) allowed the determination of viable treated cells compared to untreated controls. IC_{50} values (concentrations which caused 50% of cell death) were determined as the average of triplicates and their standard deviations were calculated. Stock concentrations for all metal complexes used in these biological assays were adjusted/verified after ICP-OES metal quantification.

10. Cellular Pt accumulation

Approximately 5×10^6 A2780 and A549 cells were plated in 100 mm Petri dishes and allowed to attach for 24 h. Then the plates were exposed to complexes at 2 μM. Additional plates were incubated with medium alone as a negative control. After 1 h of incubation in the dark at 310

K, the cells were rinsed with cold PBS and harvested by trypsinisation. The number of cells in each sample was counted manually using a haemocytometer. Then the cells were centrifuged to obtain the whole cell pellet. Cell pellets were digested in concentrated 72% v/v nitric acid (200 μL) in an oven at 343 K overnight and diluted by 3800 μL Milli-Q water to obtain the final solution. All experiments were conducted in triplicate.

11. ROS generation

Approximately 1×10^5 A549 cells were seeded in glass-bottom cell culture dishes (CELLview) and cultured 24 h for attachment. Duplicate plates were treated with complexes at 2 μM in the dark for 1 h at 310 K, then one was irradiated with blue light (465 nm), while the other was kept in the dark. Supernatant was removed and cells were incubated with DCFH-DA (20 μM) for 40 min then washed by HBSS before measurement. The fluorescence images were recorded on a fluorescence confocal microscope (LSM 880, AxioObserver, $\lambda_{ex}/\lambda_{em}$ = 488/507–611 nm).

Table S1. Crystal data and structure refinement for **2a**, **3a**, **3b** and **3c**.

Complex	**2a**	**3a**	**3b**	**3c**
CDDC number	2003123	2003125	2003126	2003124
Empirical formula	$C_{20}H_{16}N_8O_5Pt$	$C_{22.5}H_{16.75}Cl_2N_{8.25}O_{6.08}Pt$	$C_{22}H_{22}Cl_2N_8O_4Pt$	$C_{14}H_{12}Cl_4N_8O_4Pt$
Formula weight	643.50	766.01	728.46	693.21
Temperature/K	150(2)	150(2)	150(2)	150(2)
Crystal system	triclinic	trigonal	triclinic	triclinic
Space group	P-1	R-3	P-1	P-1
a/Å	7.6941(3)	37.7424(3)	9.5857(2)	7.2235(2)
b/Å	9.2101(3)	37.7424(3)	9.73231(19)	8.4944(2)
c/Å	16.4767(6)	9.84140(10)	15.2124(2)	10.0233(3)
α/°	86.004(3)	90	98.1190(14)	105.343(3)
β/°	84.751(3)	90	103.9082(15)	95.673(3)
γ/°	67.335(4)	120	104.4649(18)	108.477(3)
Volume/Å^3	1072.15(7)	12140.8(2)	1302.96(4)	551.19(3)
Z	2	18	2	1
ρ_{calc}g/cm^3	1.993	1.886	1.857	2.088
μ/mm^{-1}	6.596	12.037	12.352	6.888
F(000)	620.0	6663.0	708.0	330.0
Crystal size/mm^3	0.2 × 0.1 × 0.04 yellow block	0.3 × 0.1 × 0.08 yellow block	0.16 × 0.06 × 0.01 colourless block	0.3 × 0.18 × 0.005 colourless block
Radiation	MoKα (λ = 0.71073)	CuKα (λ = 1.54184)	CuKα (λ = 1.54184)	MoKα (λ = 0.71073)
2Θ range for data collection/°	4.796 to 62.186	8.114 to 146.702	9.608 to 156.79	5.324 to 62.12
Index ranges	-11 ≤ h ≤ 11, -12 ≤ k ≤ 13, -23 ≤ l ≤ 23	-46 ≤ h ≤ 46, -46 ≤ k ≤ 42, -10 ≤ l ≤ 12	-12 ≤ h ≤ 12, -11 ≤ k ≤ 12, -19 ≤ l ≤ 19	-10 ≤ h ≤ 10, -12 ≤ k ≤ 12, -13 ≤ l ≤ 14
Reflections collected	62932	49969	47810	33847
Independent reflections	6486 [R_{int} = 0.0564, R_{sigma} = 0.0292]	5410 [R_{int} = 0.0711, R_{sigma} = 0.0284]	5513 [R_{int} = 0.0596, R_{sigma} = 0.0267]	3261 [R_{int} = 0.0678, R_{sigma} = 0.0338]
Data/restraints/parameters	6486/4/313	5410/4/366	5513/12/334	3261/0/142
Goodness-of-fit on F^2	1.046	1.082	1.136	1.023
Final R indexes [*I*>=2*σ* (*I*)]	R_1 = 0.0225, wR_2 = 0.0475	R_1 = 0.0314, wR_2 = 0.0814	R_1 = 0.0309, wR_2 = 0.0751	R_1 = 0.0303, wR_2 = 0.0731
Final R indexes [all data]	R_1 = 0.0261, wR_2 = 0.0492	R_1 = 0.0317, wR_2 = 0.0817	R_1 = 0.0329, wR_2 = 0.0760	R_1 = 0.0308, wR_2 = 0.0737
Largest diff. peak/hole/e Å^{-3}	1.17/-1.33	1.51/-1.10	3.50/-1.55	1.66/-1.63

Table S2. Selected bond lengths (Å) and bond angles (°) for **3b** and **3c**.

**3b**		**3c**	
Pt1–O12	2.016(3)	Pt1–O10	2.006(3)
Pt1–N30	2.054(3)	Pt1–N1	2.034(3)
Pt1–N27	2.061(3)	Pt1–N7	2.047(3)
Pt1–O11	1.992(3)	N7–N8	1.223(5)
Pt1–N21	2.031(3)	N8–N9	1.141(5)
Pt1–N15	2.035(3)	O10–Pt1–O10[1]	180.0(3)
N27–N28	1.220(6)	N1–Pt1–N1[1]	180.0
N28–N29	1.143(6)	N7–Pt1–N7[1]	180.0
N31–N30	1.227(5)	N8–N7–Pt1	117.8(3)
N31–N32	1.132(6)	N7–N8–N9	174.1(4)
O11–Pt1–O12	178.76(10)	Cl12–C12–Cl1	110.1(2)
N21–Pt1–N15	177.79(13)		
N30–Pt1–N27	179.44(14)		
N28–N27–Pt1	112.7(3)		
N29–N28–N27	177.0(5)		
N31–N30–Pt1	115.2(3)		
N32–N31–N30	176.4(4)		
Cl4A–C14–Cl4B	111.5(2)		

[1] 1-X, 1-Y, 1-Z

Table S3. Selected hydrogen bonds parameters for **2a**.

**D**	**H**	**A**	**d(D-H)/Å**	**d(H-A)/Å**	**d(D-A)/Å**	**D-H-A/°**
O3	H3B	O3	0.852(4)	2.013(11)	2.854(4)	169(6)

Table S4. Photochemical decomposition products of **2a** detected by LC-MS (positive mode).

**Peak**[a]	**Formula**	**Structure**	**Calculated**	**Found**
a	$C_{12}H_{16}N_2O_8Na_3Pt_2$	$[2\{Pt^{II}(py)(OH)_2(HCOO)\}+3Na]^+$	774.99	774.99
b	$C_{19}H_{16}N_3O_4Pt$	$\{Pt^{II}(py)(cou)(CH_3CN)_2\}^+$	545.08	545.10
c	$C_{11}H_{11}N_5O_2Pt$	$\{Pt^{III}(py)_2(HCOO)(N_3)\}^+$	440.06	440.07
d	$C_{12}H_{13}N_6Pt$	$\{Pt^{II}(py)_2(N_3)(CH_3CN)\}^+$	436.08	436.08
e	$C_{10}H_7O_4$	Coumarin-3 carboxylic acid+H^+	191.03	191.29
f	$C_{20}H_{16}N_5O_5Pt$	$\{Pt^{IV}(py)_2(N_3)(OH)(cou)\}^+$	601.08	601.18
g	$C_{10}H_{10}N_8NaPt$	$[\{Pt^{II}(py)_2(N_3)_2\}+Na]^+$	460.06	460.08
h	$C_{16}H_{13}N_4O_6Pt$	$[\{Pt^{II}(py)(cou)(HCOO)(N_3)\}+2H]^+$	552.05	552.10

[a] See **Figure S20**.

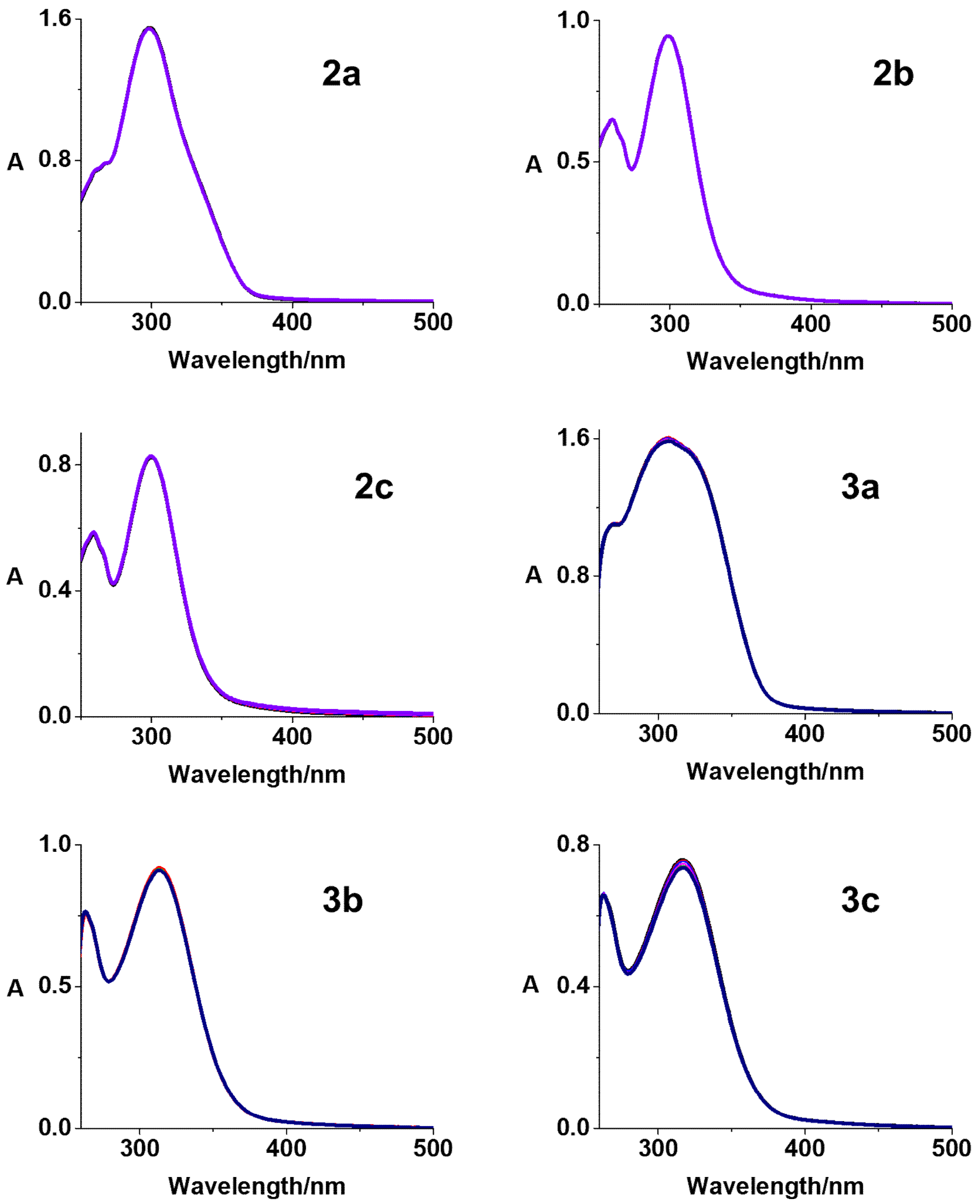

Figure S1. Dark stability of mono-functionalised complexes **2a**–**2c** (50 µM) in phenol red-free RPMI-1640 and di-functionalised complexes **3a**–**3c** (50 µM) in DMSO for 120 min monitored by UV-vis spectroscopy at 298 K. Spectra were recorded at 0, 20, 40, 90, 120 min.

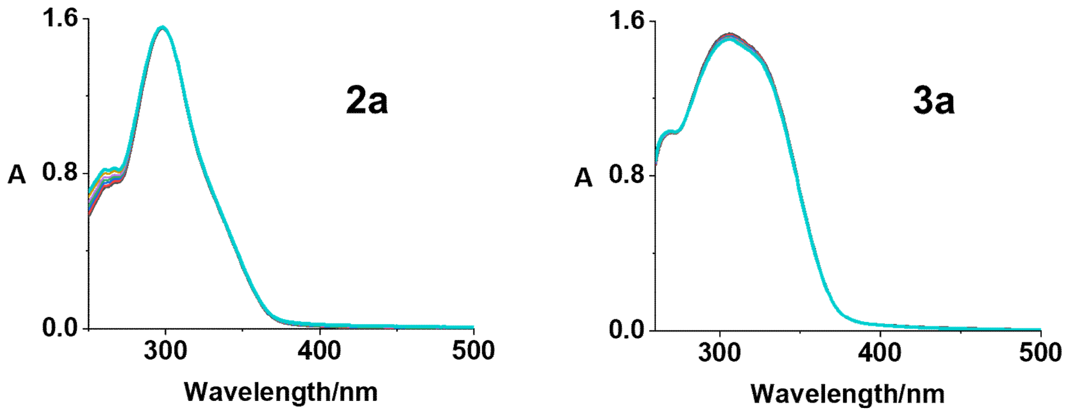

Figure S2. Dark stability in the presence of 2 mM GSH of mono-functionalised complex **2a** (50 µM) in H_2O with 5% DMSO and di-functionalised complex **3a** (50 µM) in DMSO with 5% H_2O for 120 min monitored by UV-vis spectroscopy at 298 K. Spectra were recorded at 0, 5, 20, 40, 60, 90, 120 min.

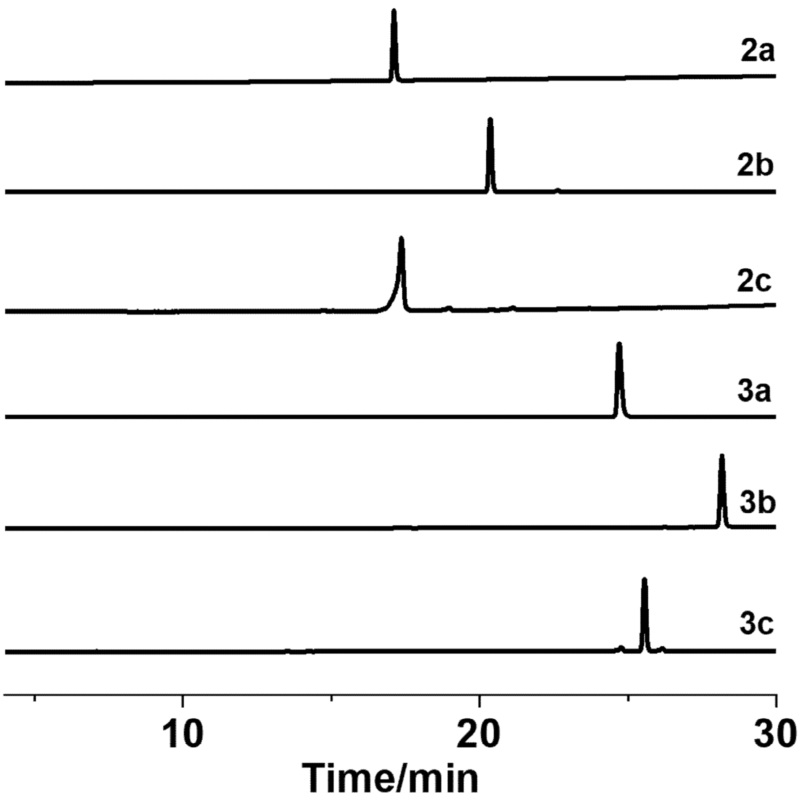

Figure S3. HPLC purity of complexes **2a**–**2c** and **3a**–**3c** with detection wavelength 254 nm (gradient 10–80% CH_3CN in 30 min).

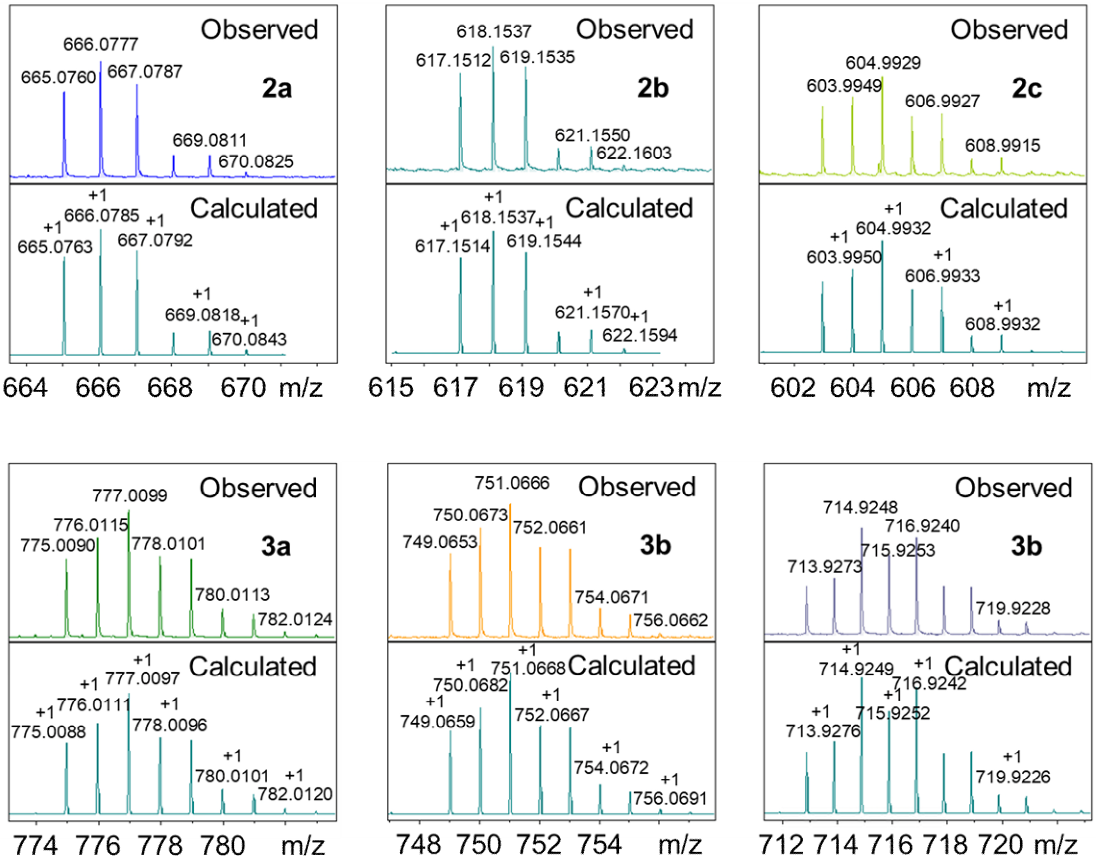

Figure S4. HR-MS of complexes **2a**–**2c** and **3a**–**3c** (observed and calculated).

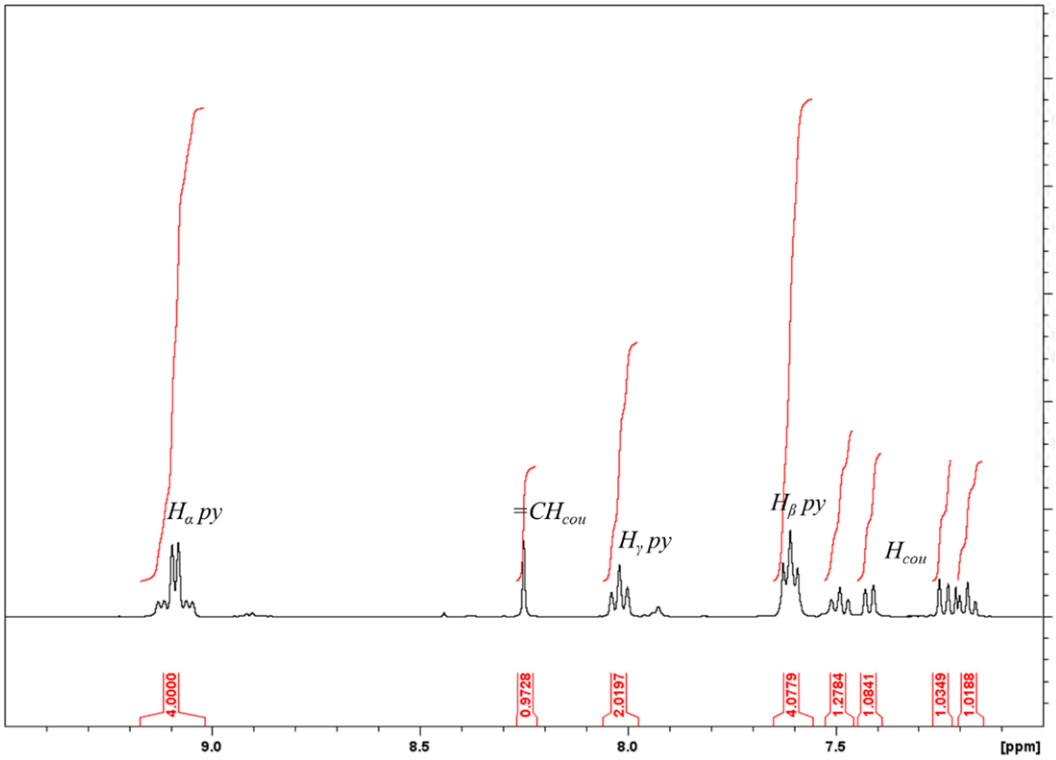

Figure S5. 400 Hz ^{1}H NMR spectrum of complex **2a** in $CDCl_3$ at 298 K.

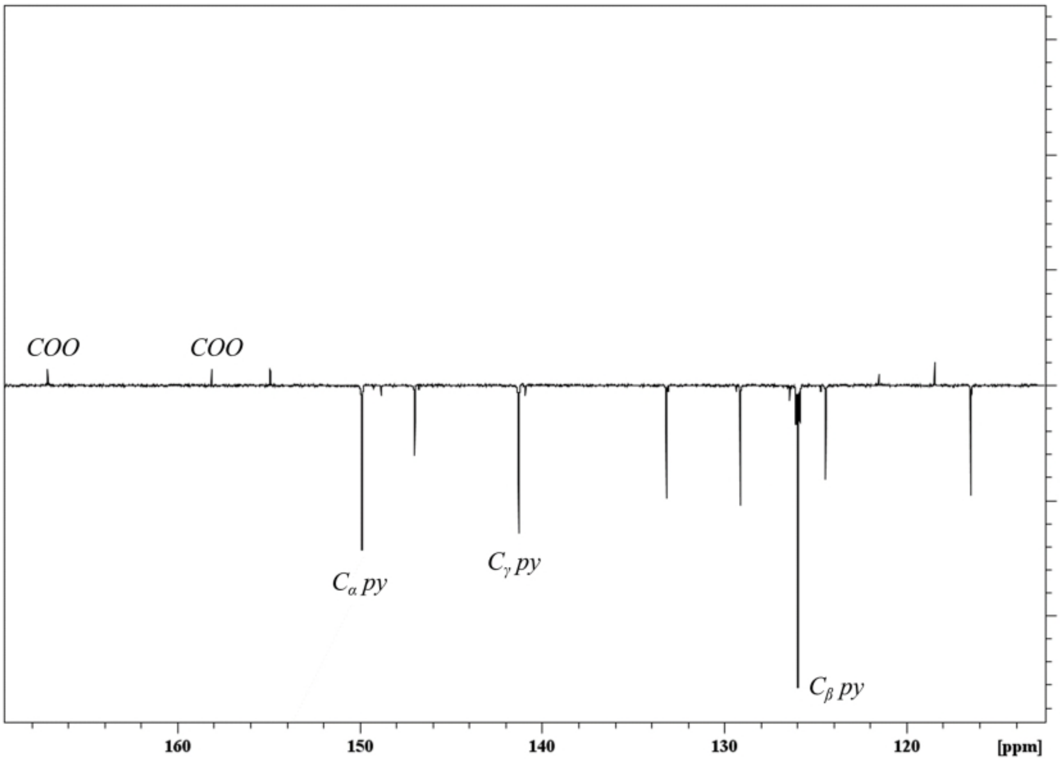

Figure S6. 125 Hz DEPT-135 ^{13}C NMR spectrum of complex **2a** in $CDCl_3$ at 298 K.

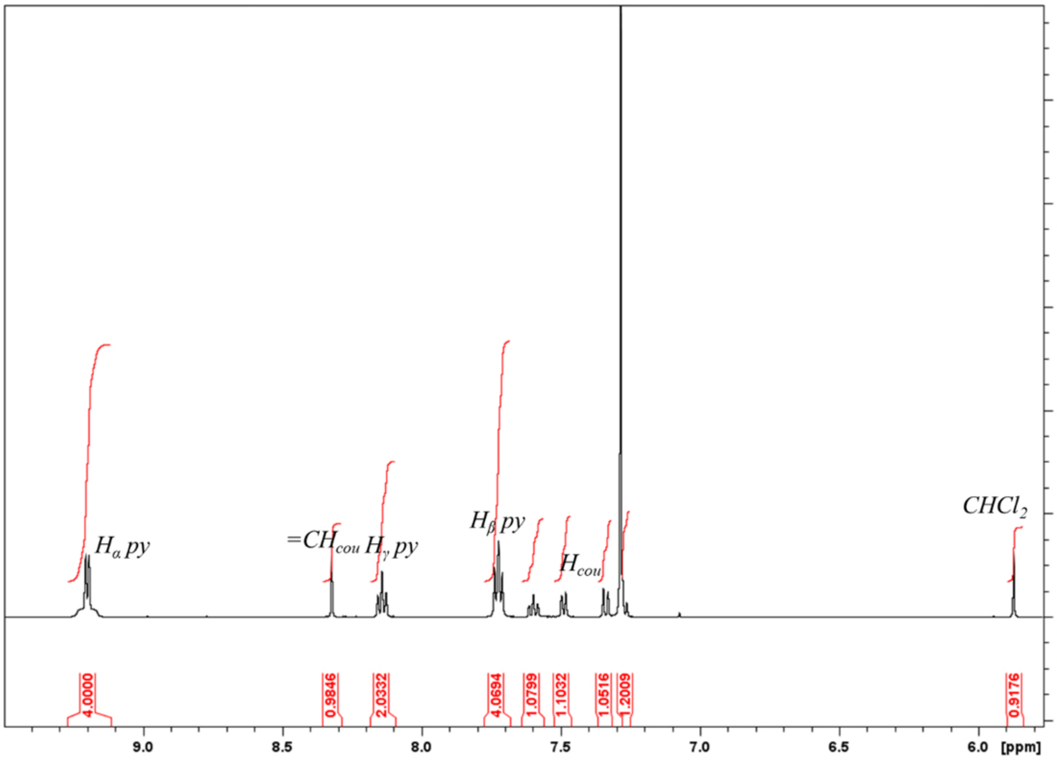

Figure S7. 500 Hz ^{1}H NMR spectrum of complex **3a** in $CDCl_3$ at 298 K.

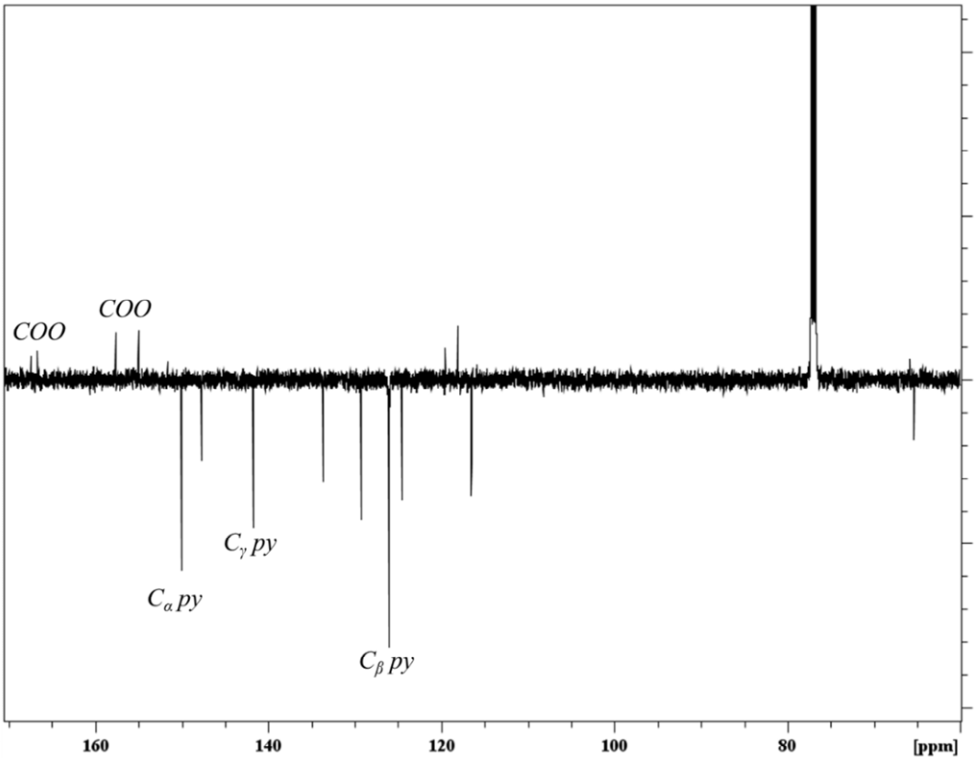

Figure S8. 125 Hz DEPT-135 ^{13}C NMR spectrum of complex **3a** in $CDCl_3$ at 298 K.

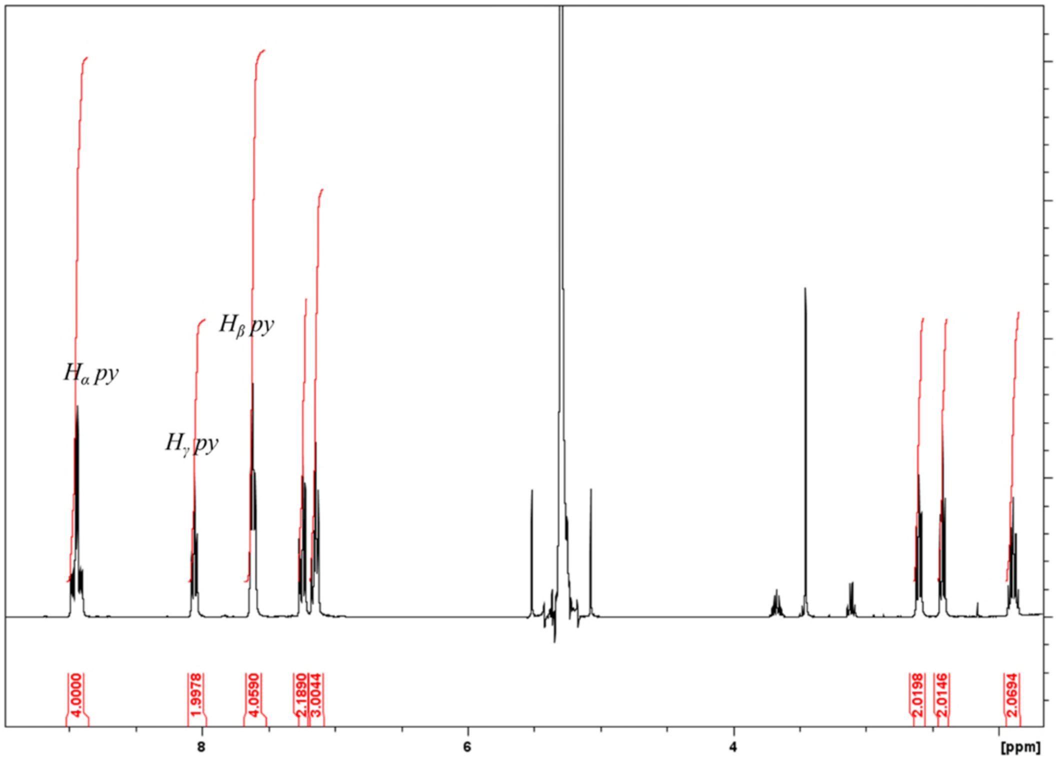

Figure S9. 400 Hz ^{1}H NMR spectrum of complex **2b** in $CDCl_3$ at 298 K.

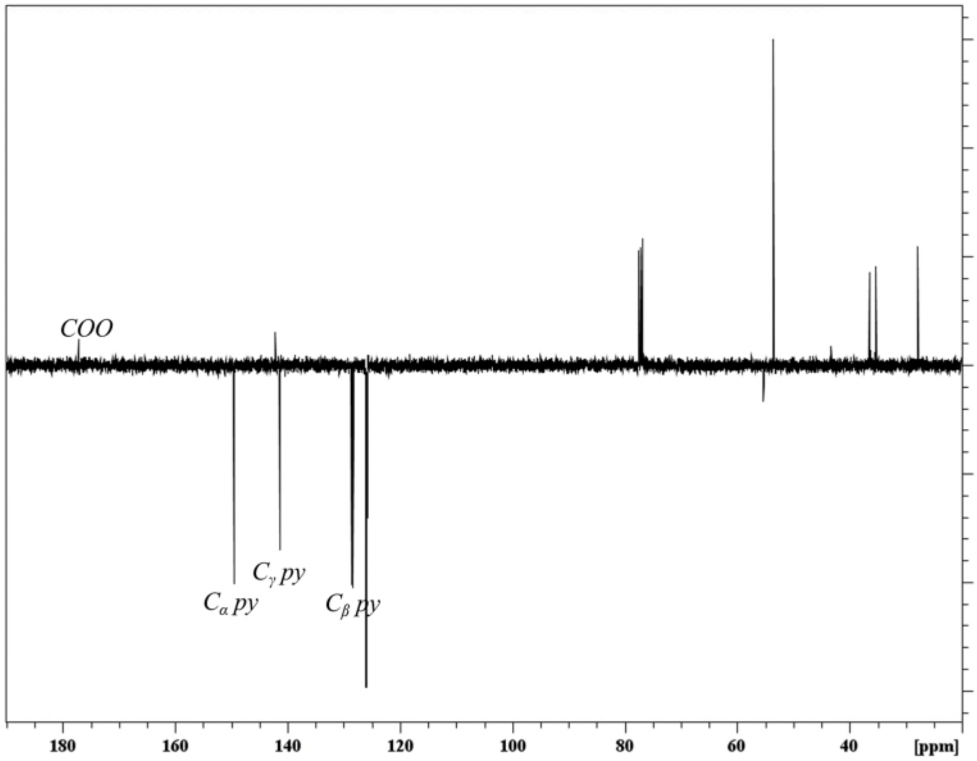

Figure S10. 100 Hz DEPT-135 ^{13}C NMR spectrum of complex **2b** in $CDCl_3$ at 298 K.

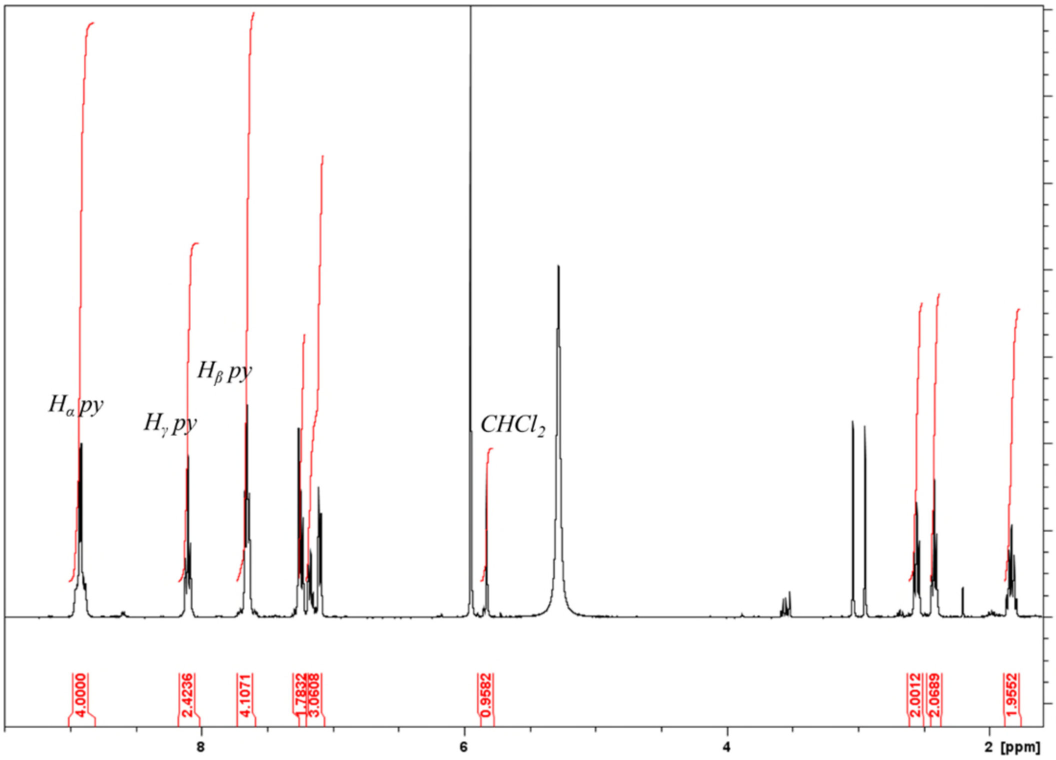

Figure S11. 400 Hz ^{1}H NMR spectrum of complex **3b** in $CDCl_3$ at 298 K.

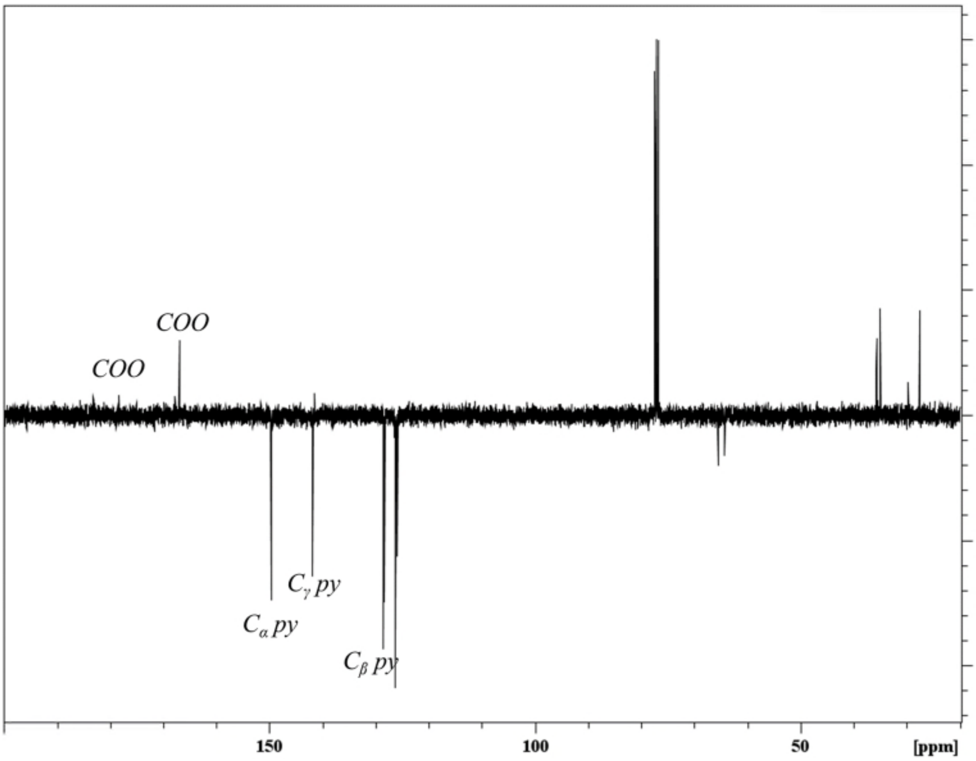

Figure S12. 100 Hz DEPT-135 ^{13}C NMR spectrum of complex **3b** in $CDCl_3$ at 298 K.

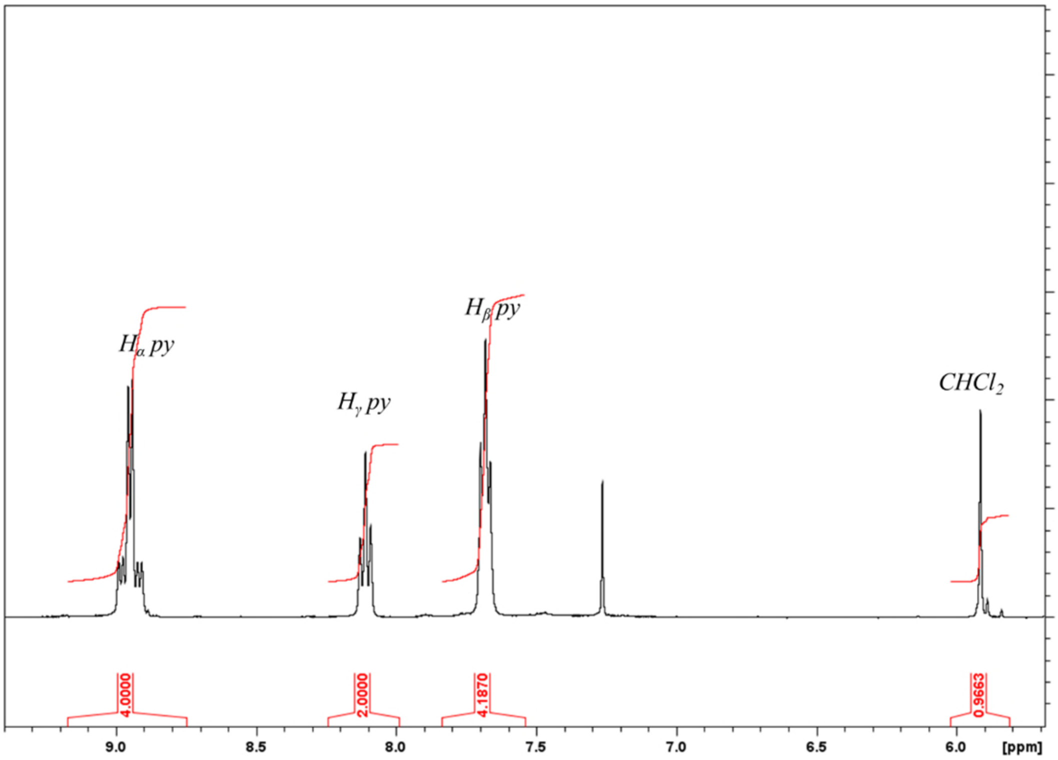

Figure S13. 400 Hz ^{1}H NMR spectrum of complex **2c** in $CDCl_3$ at 298 K.

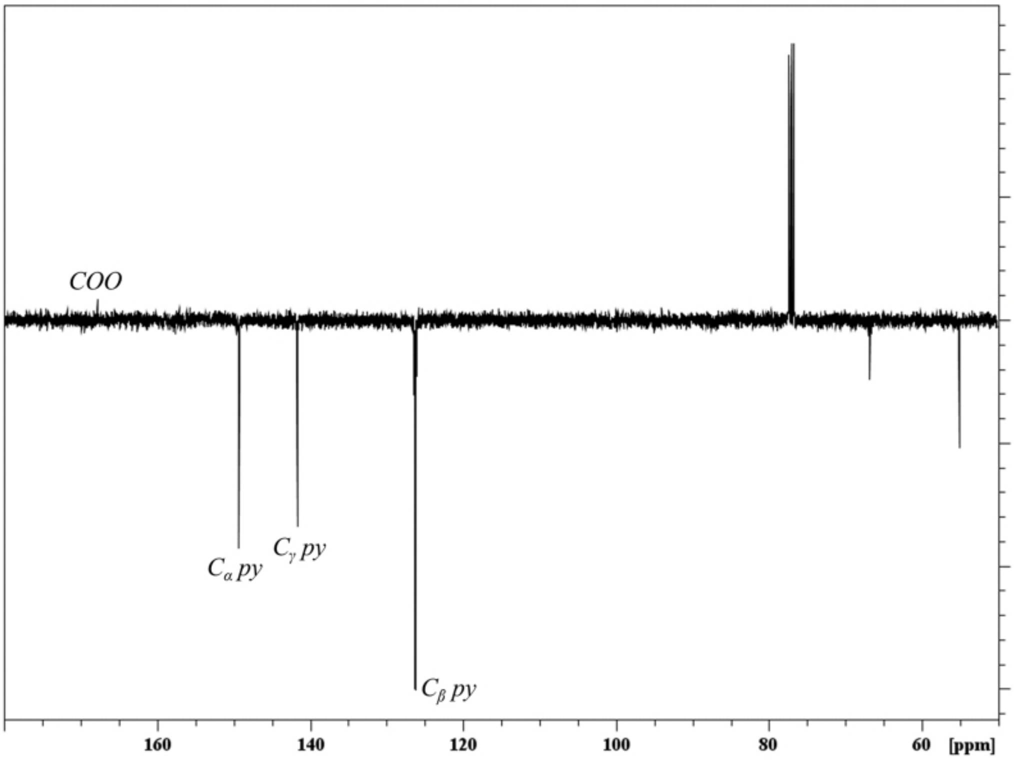

Figure S14. 100 Hz DEPT-135 ^{13}C NMR spectrum of complex **2c** in $CDCl_3$ at 298 K.

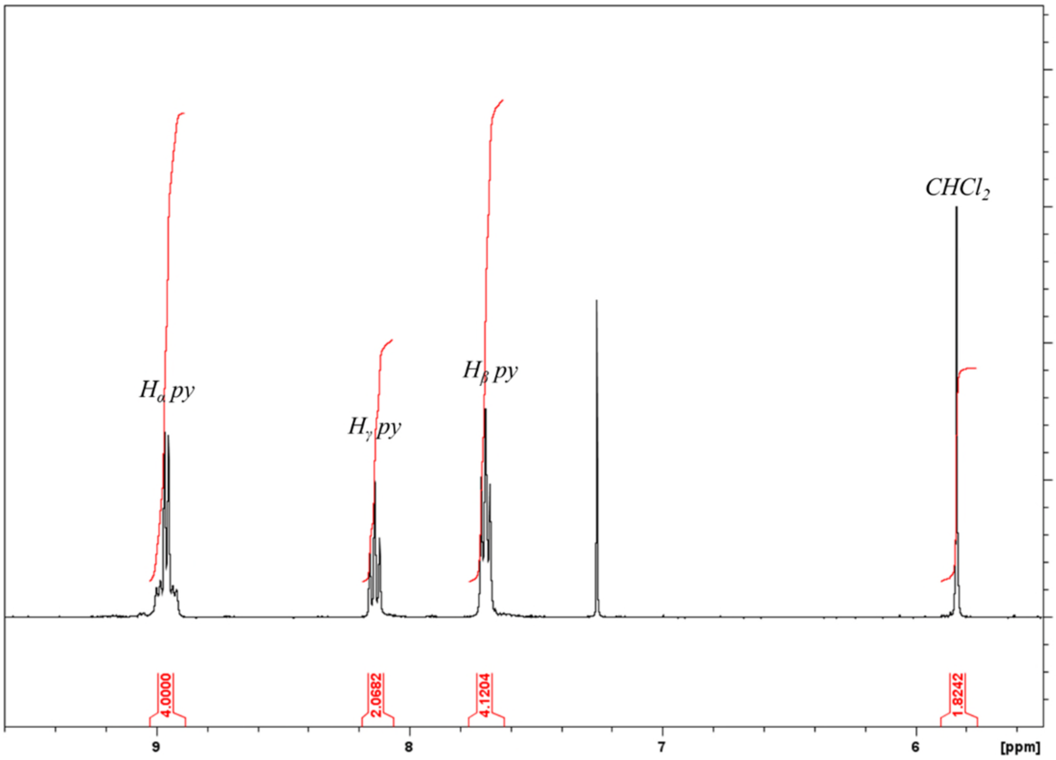

Figure S15. 400 Hz ^{1}H NMR spectrum of complex **3c** in $CDCl_3$ at 298 K.

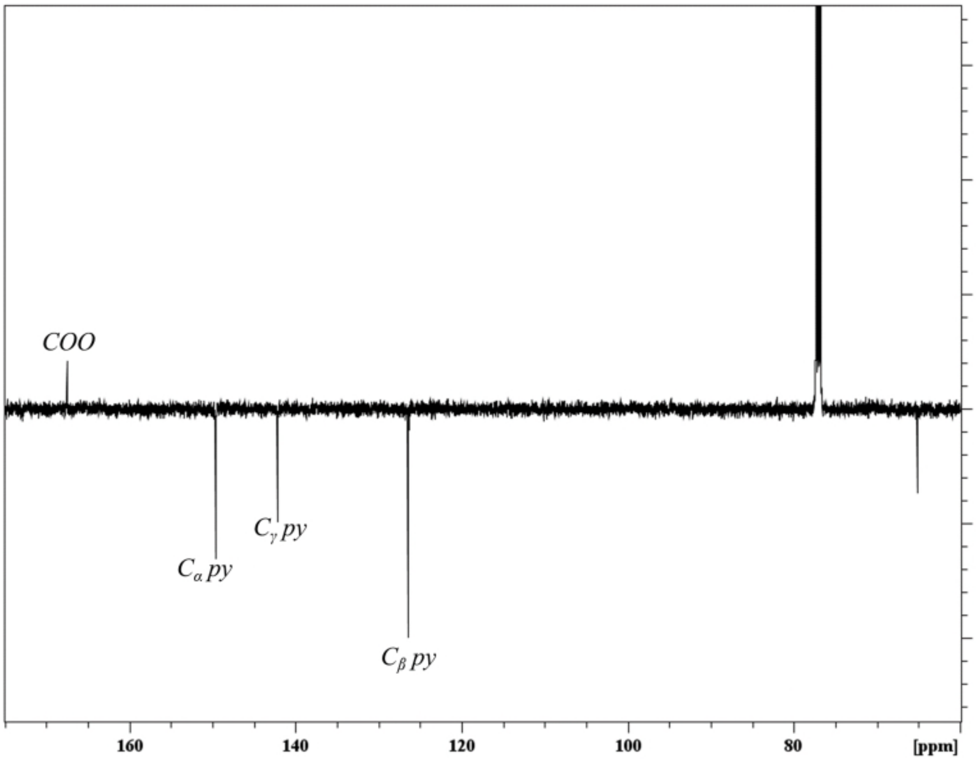

Figure S16. 125 Hz DEPT-135 ^{13}C NMR spectrum of complex **3c** in $CDCl_3$ at 298 K.

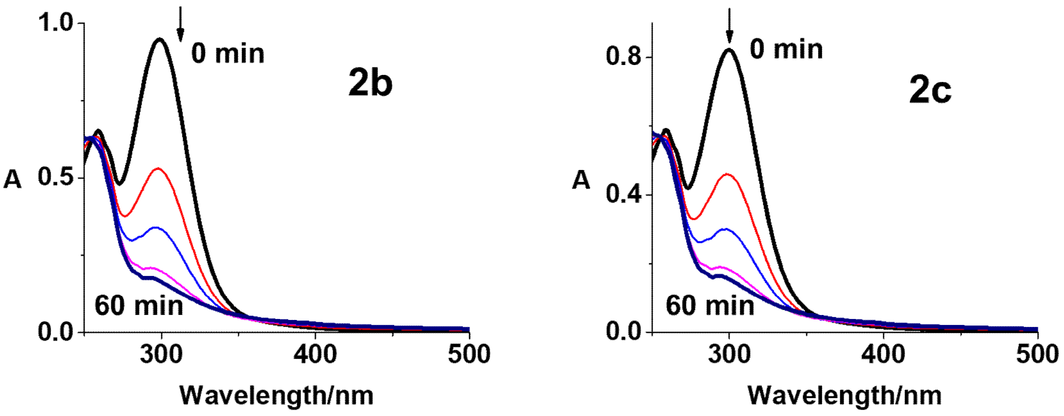

Figure S17. Photochemical decomposition of mono-functionalised complexes **2b** and **2c** in RPMI-1640 upon irradiation with blue light (420 nm, 60 min) monitored by UV-vis spectroscopy. Spectra recorded at 0, 5, 10, 20, 40, 60 min.

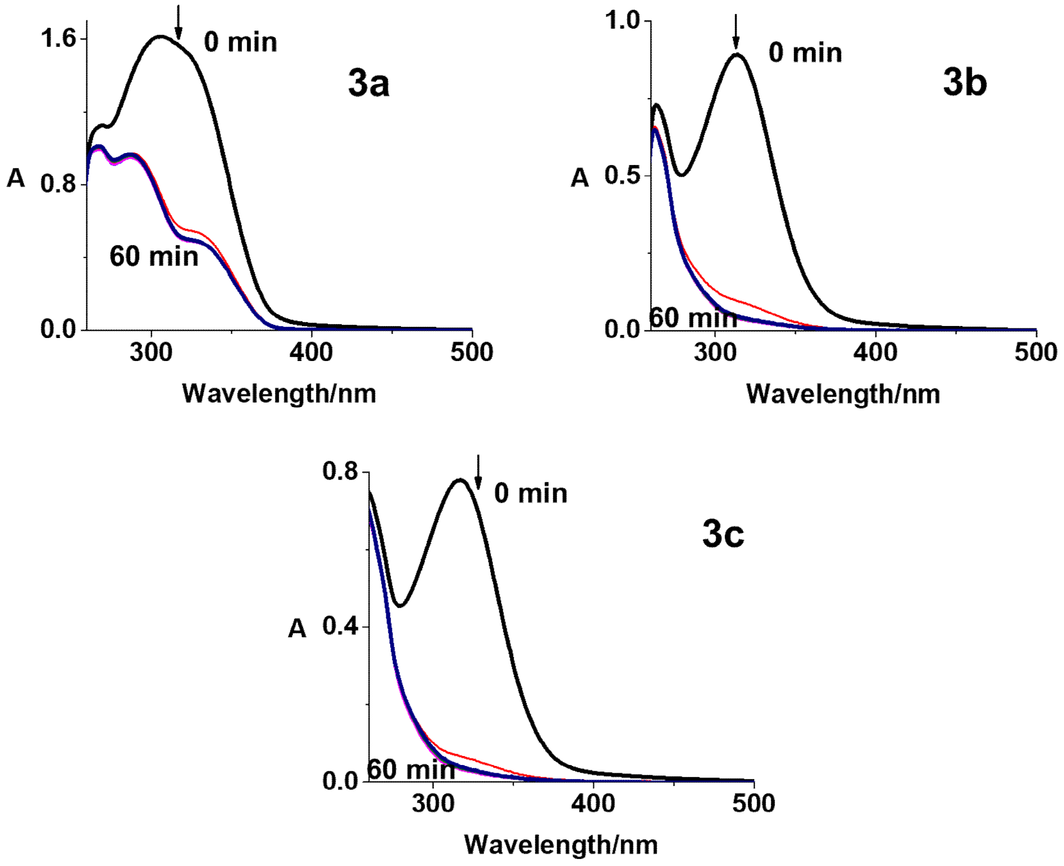

Figure S18. Photochemical decomposition of di-functionalised complexes **3a**–**3c** in DMSO upon irradiation with blue light (420 nm, 60 min) monitored by UV-vis spectroscopy. Spectra recorded at 0, 5, 10, 20, 40, 60 min.

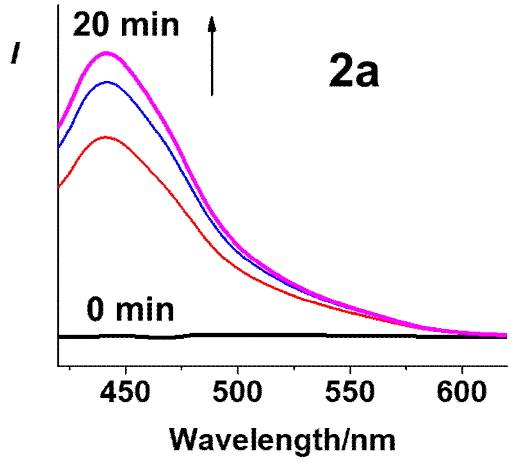

Figure S19. Fluorescence change during photochemical decomposition of complex **2a** in H_2O with 5% DMSO upon irradiation with blue light (420 nm, 20 min). Spectra recorded at 0, 5, 10, 20 min.

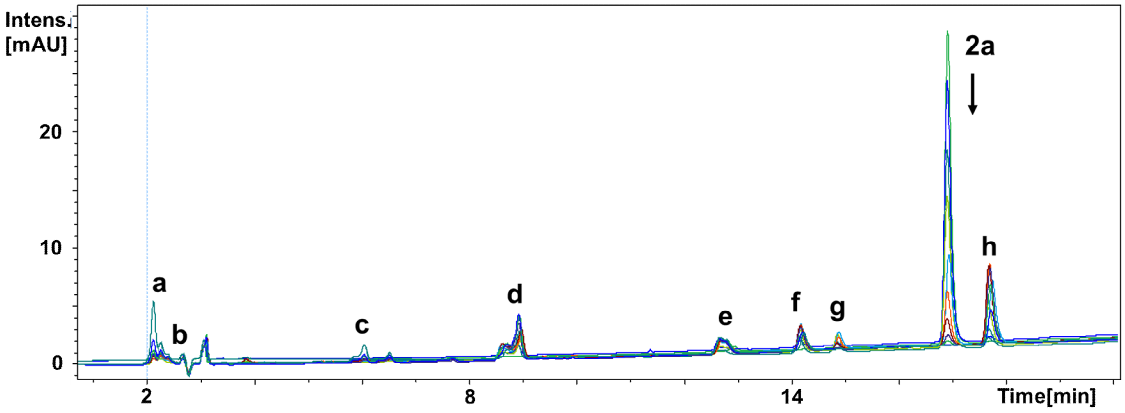

Figure S20. Photochemical decomposition of **2a** in aqueous solution monitored by HPLC upon 60 min irradiation with blue light (420 nm), possible species **a–h** are listed in **Table S4**. Spectra recorded at 0, 1, 2, 3, 5, 7, 10, 15, 20, 30, 60 min.

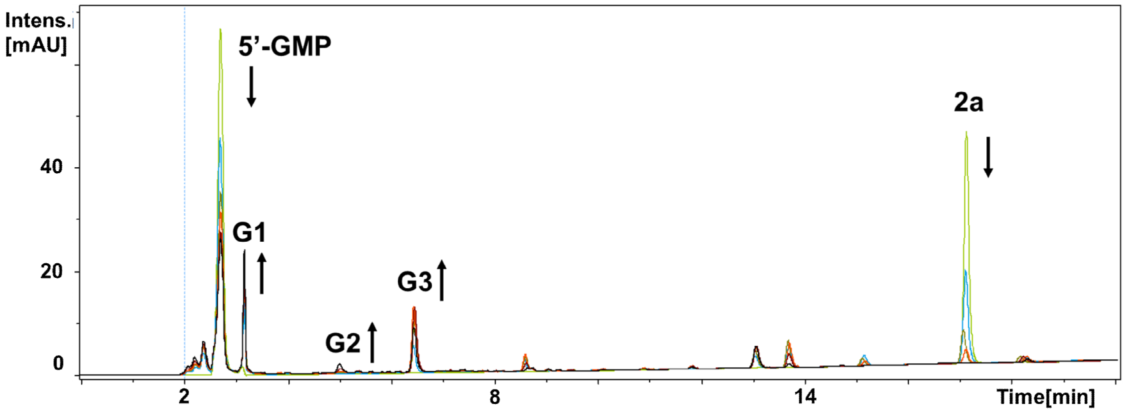

Figure S21. Photoreaction between of **2a** and 2 equiv. of 5'-GMP in aqueous solution over 60 min irradiation (420 nm) monitored by HPLC. Spectra recorded at 0, 5, 10, 15, 30, 60 min. G1: $\{Pt^{II}(CH_3CN)(py)_2(GMP\text{-}H)\}^+$ (m/z 756.16); G2: $\{Pt^{II}(HCOO)(py)_2(GMP)\}^+$ (m/z 762.10); G3: $\{Pt^{II}(N_3)(py)_2(GMP)\}^+$ (m/z 758.16).

References

S1. N. J. Farrer, J. A. Woods, L. Salassa, Y. Zhao, K. S. Robinson, G. Clarkson, F. S. Mackay and P. J. Sadler, A potent *trans*-diimine platinum anticancer complex photoactivated by visible light, *Angew. Chem., Int. Ed.*, 2010, **49**, 8905–8908.

S2. O. V. Dolomanov, L. J. Bourhis, R. J. Gildea, J. A. K. Howard, H. Puschmann, OLEX2: a complete structure solution, refinement and analysis program, *J. Appl. Crystallogr.*, 2009, **42**, 339−341.

S3. G. M. Sheldrick, SHELXT–integrated space-group and crystal-structure determination, *Acta Crystallogr., Sect. A: Found. Adv.*, 2015, **71**, 3−8.

S4. G. M. Sheldrick, Crystal structure refinement with SHELXL, *Acta Crystallogr., Sect. C: Struct. Chem.*, 2015, **71**, 3−8.

S5. V. Vichai, K. Kirtikara, Sulforhodamine B colorimetric assay for cytotoxicity screening, *Nat. Protoc.*, 2006, **1**, 1112−1116.
